# Chemistry, Applications, and Synthesis Methods of Indole Derivatives: A Comprehensive Review

**DOI:** 10.1002/tcr.202500121

**Published:** 2025-10-08

**Authors:** Raphael Silva Moratório de Moraes, Ana Beatriz Mestre Botelho, Gabriel Tavares de Almeida Pinto, Searitha Couto Rodrigues, Maria Tereza Miranda Martins, Deivid Lucas Alves Soares, Camille Cardoso Cruz, Aline de Almeida Pinto, Flaviana Rodrigues Fintelman Dias, Patricia Dias Fernandes, Anna Claudia Cunha

**Affiliations:** ^1^ Instituto de Química, Departamento de Química Orgânica, Programa de Pós‐Graduação em Química Universidade Federal Fluminense Niterói Rio de Janeiro 24020‐141 Brazil; ^2^ Instituto de Ciências Biomédicas, Laboratório de Farmacologia da Dor e da Inflamação, Centro de Ciências da Saúde Universidade Federal do Rio de Janeiro Rua Cesar Pernetta 30 Cidade Universitária Rio de Janeiro 21941‐590 Brazil

**Keywords:** biological activities, biosynthesis, indoles, material chemistry, synthetic methods

## Abstract

Indole and its derivatives represent a crucial class of heterocyclic compounds with broad applications in pharmaceuticals, agrochemicals, and materials science. The indole core is a fundamental structural motif in numerous biologically active natural products, including alkaloids, as well as synthetic molecules exhibiting diverse pharmacological properties such as anticancer, antimicrobial, anti‐inflammatory, and antiviral activities. This review concisely addresses the biosynthesis of the indole nucleus and highlights several noteworthy derivatives, including tryptophan, indigo, and indole‐3‐carbinol, among others. It explores the chemistry of indole derivatives, emphasizing their multidisciplinary relevance, and substantial impact across various fields of scientific research. Furthermore, the review discusses both classical and contemporary synthetic methodologies for constructing the indole framework, with an emphasis on sustainable approaches aligned with green chemistry principles.

## Graphical abstract

Indole and its derivatives represent a vital class of heterocycles with broad applications in pharmaceuticals, agrochemicals, and materials science. This review explores their applications, biosynthesis, key derivatives such as tryptophan, and presents an overview of both classical and modern synthetic methodologies, with a focus on sustainable approaches and interdisciplinary relevance.

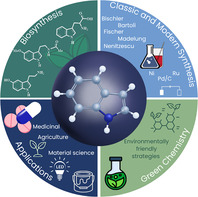

## 1 Introduction

Indole is a planar^[^ ^1^ ^]^ *N*‐heterocyclic compound^[^ ^2^ ^]^ with the chemical formula C_8_H_7_N.^[^ ^3^ ^]^ Indole is a solid at room temperature and occurs naturally in human feces, contributing to its characteristic odor.^[^ ^3^, ^4^ ^]^ It is classified as an aromatic compound, featuring a bicyclic structure composed of a six‐membered benzene ring fused to a five‐membered pyrrole ring.^[^ ^5^ ^]^ It is very weakly basic (pKa of protonated indole = −3.6) due to the involvement of the lone pair of the incorporated nitrogen in the resonance of the aromatic bicyclic structure.^[^ ^6^ ^]^

The indole compound readily participates in various chemical reactions due to its versatile bonding sites, similar to pyrrole. Indole is reactive at four key positions: the C3 carbon atom, the *N‐*1 nitrogen atom, the C2—C3 π‐bond, and the C2—N sigma bond. Indole can be protonated at the C3 position more readily than at the nitrogen atom when exposed to strong acids such as hydrochloric acid. This preference is due to the enamine‐like reactivity of the region outside the benzene ring. Besides that, its aromaticity delocalizes the nitrogen lone pair, making it unavailable for protonation.^[^ ^7^ ^]^

**Figure 1:**
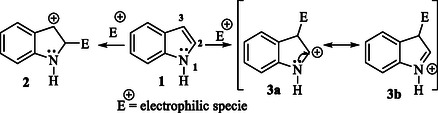
Electrophilic aromatic substitution of indole.^[^ ^186^ ^]^

Additionally, indole undergoes cycloaddition reactions, with the C2‐C3 π‐bond being particularly reactive. Cycloaddition at the C2—N sigma bond is also observed, albeit less frequently.^[^ ^8^ ^]^ The C3 position of indole is 10^13^ times more reactive toward electrophilic aromatic substitution than benzene.^[^ ^9^ ^]^ Indole **1** is more reactive than benzene because the N atom increases the ring electron density (**Figure** **1**). The preferred site for electrophilic substitution is C3 rather than C2, in contrast to pyrrole. The electrophilic attack at position C3 is favored because the positive charge can be delocalized without involving the fused benzene ring **2** and **3a–b**.^[^ ^10^ ^]^

**Scheme 1:**
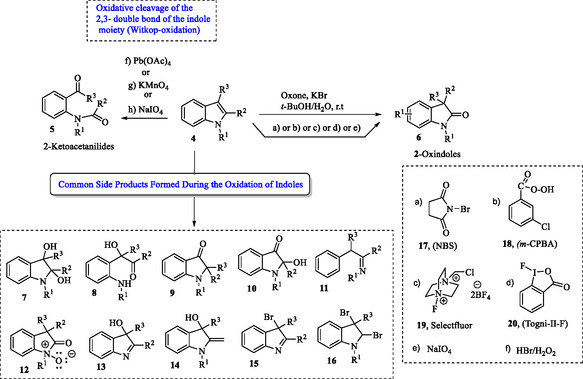
Oxidative strategies for the structural diversification of indole derivatives.

The electron‐rich nature of indoles makes them highly susceptible to oxidation under diverse conditions. However, oxidative transformations (**5** ← **4** → **6**) frequently lead to complex product mixtures **7–16** due to competing reactions at the nitrogen, C2, and C3 positions, along with potential rearrangements and over‐oxidation.^[^ ^11^ ^]^ Some examples of oxidizing agents used for the oxidation of indoles include Oxone (KHSO_5_‐1/2KHSO_4_‐1/2K_2_SO_4_), *N*‐bromosuccinimide (**17**, NBS), *meta*‐chloroperoxybenzoic acid (**18**, *m*‐CPBA), chloromethyl‐4‐fluoro‐1,4‐diazoniabicyclo[2.2.2]octane *bis*(tetrafluoroborate) (**19**, Selectfluor, F‐TEDA‐BF_4_), 1‐trifluoromethyl‐1,2‐benziodoxol‐3(1*H*)‐one (**20,** Togni‐II‐F), sodium periodate (NaIO_4_),^[^ ^11^ ^]^ and (HBr/H_2_O_2_),^[^ ^12^ ^]^ as represented in the **Scheme** **1**.

**Scheme 2:**
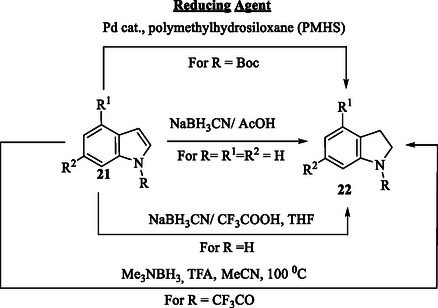
Reductive transformation of indole **21** derivatives into indolines **22**.

Indole **21** can be reduced to indoline **22** through catalytic hydrogenation or various other reduction methods, including treatment with sodium cyanoborohydride, zinc powder, tin, or zinc amalgam in hydrochloric acid, and sodium in ammonia, among others (**Scheme** **2**). The indoline scaffold **22** is widely present in natural products and has recently attracted considerable attention as a key structural motif in drug design.^[^ ^13^, ^14^, ^15^ ^]^

## 2 Tryptophan: A Vital Indole Derivative in Biological Systems

**Figure 2:**
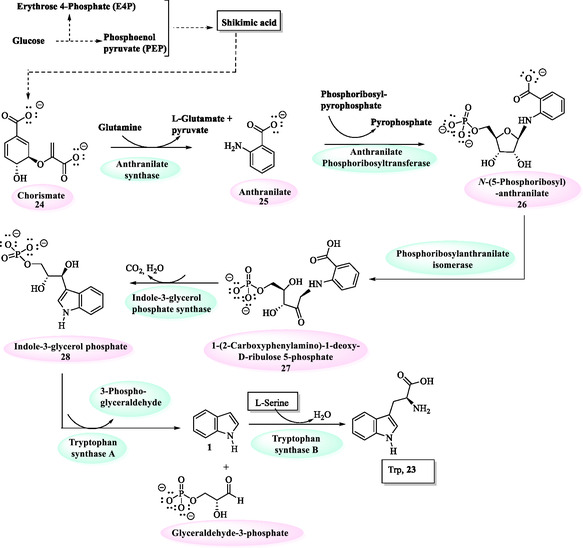
Synthesis of tryptophan **23** from chorismate **24**.

Indole derivatives are widely distributed in nature and play a vital role in numerous physiological processes. A notable example is tryptophan (Trp) (2‐amino‐3‐(1*H*‐indol‐3‐yl)propanoic acid, **23**) (**Figure** **2**), an essential amino acid distinguished by the presence of an indole moiety in its side chain.

Trp **23** biosynthesis occurs via the shikimate pathway (Figure 2), also known as the chorismate biosynthesis pathway, which converts two key metabolites, phosphoenolpyruvate (PEP) from glycolysis and erythrose 4‐phosphate (E4‐P) from the nonoxidative phase of the pentose phosphate pathway, into chorismate **24**. Chorismate then undergoes a series of enzymatic transformations, ultimately leading to the formation of Trp **23**.^[^ ^16^ ^]^

The biosynthesis of Trp **23** via the chorismate pathway is fundamental to the metabolic architecture of plants, bacteria, and fungi, as it provides an essential route for the formation of aromatic amino acids and key secondary metabolites. In humans, this amino acid is not produced by the body (endogenously) and, therefore, must be obtained through the diet. The biosynthetic pathway of this aromatic indole amino acid leads to the formation of valuable compounds with significant importance in the development of herbicides and antibiotics. Furthermore, Trp **23** catabolism significantly influences the microenvironment, playing a key role in modulating cancer immune cell responses.^[^ ^17^ ^]^

**Figure 3:**
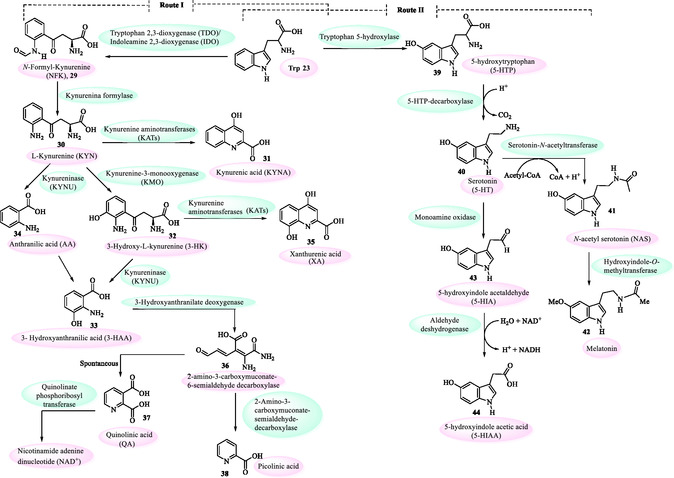
Tryptophan oxidation through the kynurenine pathway (Route I); and Tryptophan metabolism via serotonin synthesis and degradation pathway (Route II).

Tryptophan (**23**, Trp) is essential for protein synthesis and serves as a precursor in multiple metabolic pathways, generating bioactive metabolites that interact with the aryl hydrocarbon receptor (AhR), playing a crucial role in regulating inflammatory and immune responses.^[^ ^18^, ^19^ ^]^ Among these pathways,^[^ ^20^, ^21^, ^22^, ^23^ ^]^ three are particularly significant, with the kynurenine pathway accounting for more than 90% of dietary Trp metabolism. This route produces several biologically active metabolites, including L‐kynurenine **30** (KYN), kynurenic acid **31** (KYNA), 3‐hydroxykynurenine **32** (3‐HK), 3‐hydroxyanthranilic acid **33** (3HAA), anthranilic acid **34** (AA), and quinolinic acid **37**, among others (**Figure** **3**, Route I).

This pathway is regulated by the enzymes indoleamine 2,3‐dioxygenase (IDO) and tryptophan 2,3‐dioxygenase (TDO), and is activated by proinflammatory stimuli. Dysregulation of the kynurenine pathway has been associated with cancer, neurodegenerative disorders, and psychiatric diseases.^[^ ^24^, ^25^ ^]^

**Figure 4:**
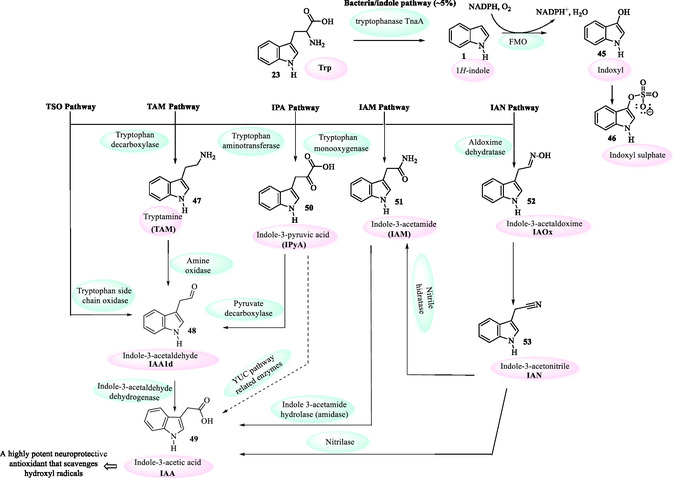
Tryptophan metabolism by human gut microbiota.^[^ ^26^ ^]^

Approximately 5% of dietary tryptophan is metabolized via the indole pathway by specific intestinal commensals, particularly certain *Escherichia coli* and *Bacteroides* strains. The biosynthetic process leads to the production of several indole derivatives **(45–53)**, with particular emphasis on the neurotransmitter tryptamine **47** and indole‐3‐acetic acid (IAA, **49**) (**Figure** **4**), the principal natural auxin that acts as an essential regulator of growth and development in plants (Section 4.1). Notably, although gut microbes produce IAA as a metabolic byproduct, its biosynthesis is more thoroughly characterized in plant‐associated *rhizobacteria*.^[^ ^26^, ^27^ ^]^

The remaining tryptophan is utilized in the 5‐hydroxyindole pathway (also known as the serotonin pathway), where it is converted into neurotransmitter serotonin **40** (5‐hydroxytryptamine or 5‐HT) and subsequently hormone melatonin **42** (*N*‐acetyl‐5‐methoxytryptamine)^[^ ^28^ ^]^ in the gut and brain, supporting key neurological and physiological functions (Figure 3, Route II).

The metabolism of serotonin **40** (5‐HT) is primarily mediated by monoamine oxidase (MAO), which catalyzes its conversion into 5‐hydroxyindoleacetaldehyde **43** (5‐HIA). This intermediate is then further metabolized by mitochondrial aldehyde dehydrogenase (ALDH) into 5‐hydroxyindole acetic acid **44** (5‐HIAA), which is predominantly excreted in the urine.^[^ ^29^ ^]^

## 3 Importance of Indole Derivatives in Medicinal Chemistry

**Figure 5:**
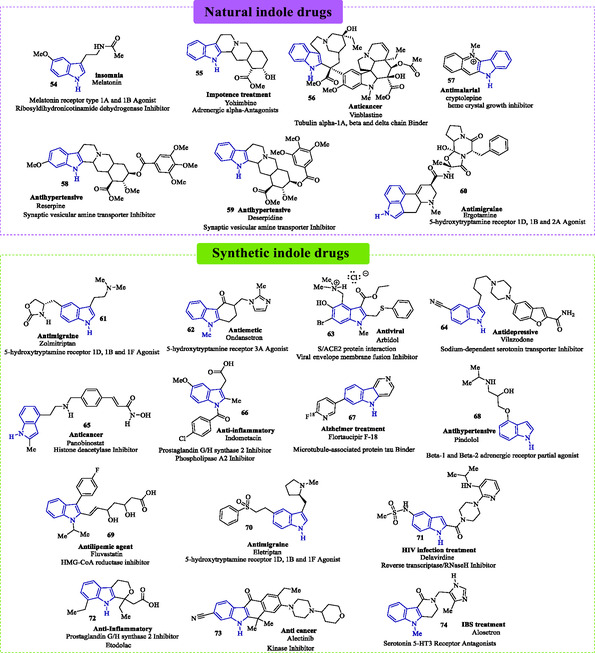
Examples of indole‐based drugs **54–74** and their respective mechanisms of action.

Numerous indole‐based drugs have shown therapeutic potential against diseases such as cancer, malaria, and other medical conditions.^[^ ^30^ ^]^ In the pharmaceutical field, substituted indoles are considered privileged scaffolds due to their ability to bind with high affinity to a broad spectrum of biological targets.^[^ ^1^, ^31^ ^]^ **Figure** **5** shows some examples of indole‐based drugs **54–74** along with their mechanisms of action.^[^ ^32^, ^33^, ^34^, ^35^, ^36^, ^37^, ^38^, ^39^, ^40^, ^41^, ^42^, ^43^, ^44^, ^45^, ^46^, ^47^ ^]^

Serving as a crucial scaffold for drug discovery, indole enables the development of therapeutically relevant compounds. By strategically incorporating functional groups or pharmacophores, researchers aim to optimize indole derivatives to enhance biological activity, refine drug‐like properties, and achieve greater selectivity for specific molecular targets.

**Figure 6:**
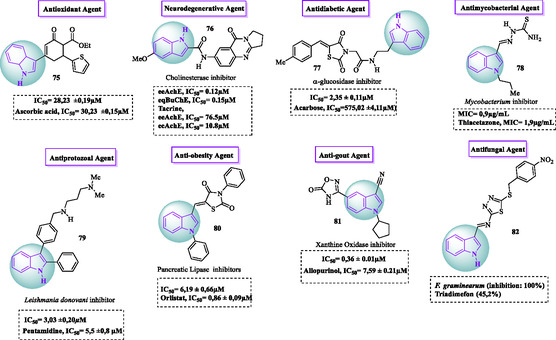
Examples of nondrug indole compounds **75–82** with diverse biological activities.

Numerous nondrug substances reported in the literature have expanded the arsenal of biologically active indole compounds.^[^ ^48^ ^]^ The **Figure** **6** presents examples of indole‐based compounds with diverse biological activities, including antioxidant **75**,^[^ ^49^ ^]^ neuroprotective **76**,^[^ ^50^ ^]^ antidiabetic **77**,^[^ ^51^ ^]^ antimycobacterial **78**,^[^ ^52^ ^]^ antiprotozoal **79**,^[^ ^53^ ^]^ antiobesity **80**,^[^ ^54^ ^]^ antigout **81**,^[^ ^55^ ^]^ and antifungal **82** ^[^ ^56^ ^]^ properties, distinct from those found in pharmaceutical products (Figure 6).

## 4 Other Applications for Indole Compounds

Indole compounds are remarkably versatile and have found applications in a wide range of fields beyond their well‐established pharmaceutical uses. Here are some notable areas where these compounds play a significant role.

### 4.1 Indoles as Plant Growth Regulators

IAA **49** (Figure 4) is one of the most abundant phytohormones synthesized by numerous rhizobacterial species that plays a pivotal role in facilitating plant colonization and stimulating plant growth.^[^ ^57^, ^58^ ^]^

The microbial biosynthesis of IAA **49** can be classified into tryptophan‐dependent (Figure 4) and tryptophan‐independent pathways.^[^ ^26^ ^]^ In the tryptophan‐dependent pathways of microorganisms, various intermediate metabolites are involved. Current research categorizes these pathways into five main types (Figure 4): the indole‐3‐acetamide (IAM) pathway, the indole‐3‐pyruvic acid (IPA/IPyA) pathway, the indole‐3‐acetonitrile (IAN) pathway, the tryptamine (TAM) pathway, and the tryptophan side‐chain oxidase (TSO) pathway.^[^ ^26^ ^]^

**Figure 7:**
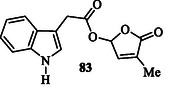
Indole with dual activity: promoting seed germination while inhibiting embryonic root growth postgermination.

Indole‐3‐acetonitrile (IAN) **53** (Figure 4) has been related as a highly effective plant growth regulator, exhibiting 10 times the efficacy of IAA.^[^ ^59^ ^]^ The introduction of the 3‐methylfuran‐2(5*H*)‐one moiety into the carboxylic acid portion of IAA led to the formation of compound **83** (**Figure** **7**), which exhibited dual activity by promoting seed germination while simultaneously inhibiting embryonic root growth postgermination.^[^ ^59^ ^]^

### 4.2 Indole‐3‐Carbinol and its Dimeric Metabolite: Biosynthesis and Therapeutic Potential

**Scheme 3:**
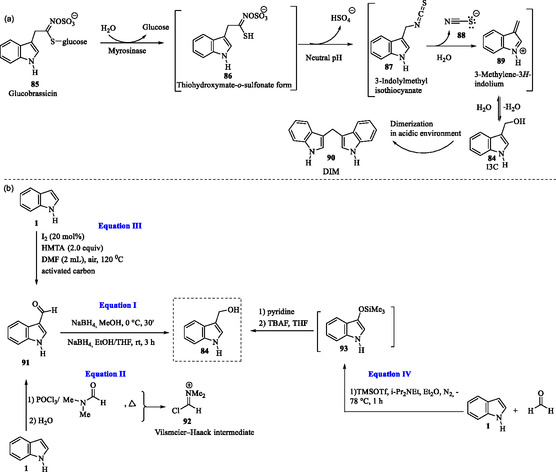
a) Biosynthesis of I3C **84** starting from glucobrassicin **85** and its dimeric metabolite DIM **90** and b) Synthetic routes for the preparation of indole‐3‐carbinol **84** (I3C).

Indole‐3‐carbinol **84** (I3C, C_9_H_9_NO) is a bioactive compound formed by the hydrolysis of the glucosinolate glucobrassicin **85** (**Scheme** **3**a), which is naturally abundant in cruciferous vegetables such as broccoli, cabbage, cauliflower, and brussels sprouts. During this process, glucobrassicin **85** is initially hydrolyzed into an unstable thiohydroximate‐*O*‐sulfonate intermediate **86**, which undergoes elimination of a hydrogen sulfate ion, leading to the formation of an unstable 3‐indolylmethyl isothiocyanate (**87**). This intermediate further decomposes, through sequences **87** to **89**, to yield indole‐3‐carbinol (**84**) as one of its key metabolic products.^[^ ^60^ ^]^

Indole‐3‐carbinol **84** is typically unstable and undergoes acid‐catalyzed condensation in the stomach and gut, forming 3,3′‐diindolylmethane **90** (DIM).^[^ ^60^ ^]^

Indole‐3‐carbinol **84** and DIM **90** (Scheme 3a) are the focus of ongoing biomedical research and are marketed as dietary supplements, primarily for their potential anticancer, anti‐inflammatory activities and neuroprotective effects. Indole‐3‐carbinol **84** also exhibits antioxidant and antiatherogenic properties.^[^ ^60^, ^61^, ^62^, ^63^, ^64^ ^]^

Among the various synthetic methodologies for obtaining I3C **84**, those most suitable for large‐scale production involve the reduction of the corresponding aldehyde **91** using sodium borohydride (NaBH_4_) (Scheme 3b, Equation I).^[^ ^60^, ^65^ ^]^ Traditionally, the Vilsmeier–Haack reaction is widely used for the formylation of indole **1**. However, it requires a stoichiometric amount of toxic phosphorus oxychloride (POCl_3_) (Scheme 3b, Equation II), which poses environmental and safety concerns. A highly efficient and chemoselective iodine‐catalyzed protocol has been developed for the 3‐formylation of both free (N—H) and *N‐*substituted indoles, utilizing hexamethylenetetramine (HMTA) and activated carbon under ambient air conditions (Scheme 3b, Equation III). This method offers a greener alternative to traditional formylation approaches, avoiding the use of toxic reagents like POCl_3_ while maintaining high selectivity and efficiency.^[^ ^66^ ^]^

In an alternative methodology, indole **1** undergoes a Friedel–Crafts addition to formaldehyde in the presence of trimethylsilyl trifluoromethanesulfonate (TMSOTf, (CH_3_)3_3_SiO_3_SCF_3_) and a trialkylamine, yielding 3‐(1‐silyloxyalkyl)indole **93**. Subsequent neutralization with pyridine, followed by deprotection under basic conditions using tetrabutylammonium fluoride (TBAF), provides indole‐3‐carbinol **84** (Scheme 3b, Equation IV).^[^ ^60^ ^]^

### 4.3 IBA‐Loaded Microspheres for Controlled Plant Growth Regulation

**Scheme 4:**
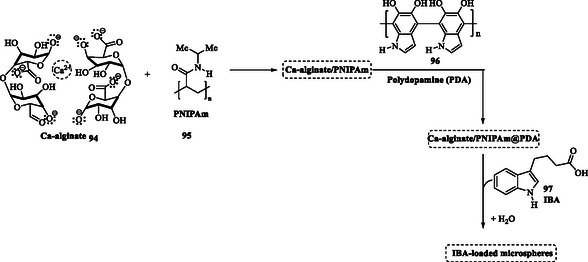
Synthesis of Ca‐alginate/Poly(*N*‐isopropylacrylamide)@polydopamine (Ca‐alginate/PNIPAm@PDA) microsphere.

Zheng et al. (2020) synthesized indole‐3‐butyric acid (IBA) loaded microspheres to develop a system that efficiently retains water and regulates the release of nutrients and agrochemicals in response to environmental changes (**Scheme** **4**). This innovative approach enhances the sustainability of agricultural practices by optimizing resource utilization and minimizing environmental impact. The Ca‐alginate/Poly(N‐isopropylacrylamide)@polydopamine (Ca‐alginate/PNIPAm@PDA) microsphere, developed for the controlled release of agrochemicals, is composed of Ca‐alginate **94**, PNIPAm) (**95**), PDA (**96**), and IBA (**97**). This combination imparts multiresponsive properties to the structure, enabling controlled release in response to various environmental stimuli such as changes in pH, temperature, and sunlight exposure. IBA **97** is a plant growth regulator widely recognized for enhancing the development of food and ornamental crops when applied to soil, cuttings, or foliage.^[^ ^67^ ^]^

This dynamic adaptability enables precise regulation of the hormone's release rate, optimizing its effectiveness in diverse agricultural conditions. This advanced controlled release system minimizes product loss through leaching and degradation, thereby enhancing IBA's effectiveness and significantly reducing environmental pollution associated with excessive agrochemical use.^[^ ^67^ ^]^

### 4.4 Indole Compounds as an Insecticidal Agents

**Figure 8:**
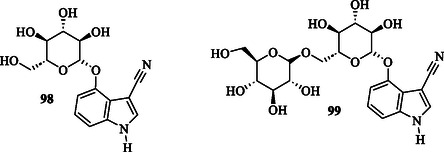
Indoles **98**,**99** as insecticidal agents.

The yellow fever mosquito, *Aedes aegypti* (*A. aegypti*), is the primary vector responsible for transmitting dengue and dengue hemorrhagic fever, both of which pose major global health challenges. Most people rely on insecticides to eliminate *A. aegypti* mosquito larvae. However, their extensive use involves heavy chemicals that can negatively impact the environment and human health. The natural indole compounds, Cappariloside A **98** and B **99** (**Figure** **8**), have demonstrated significant potential as larvicidal agents against *A. aegypti*, presenting a promising approach for sustainable vector control.^[^ ^68^ ^]^

### 4.5 Material Science of Indigo: Application and Synthesis

Indigo **100**, also known as indigotin or Vat Blue 1, is an indole derivative and one of the oldest known dyes. It is widely recognized as the pigment responsible for the iconic blue hue of denim jeans. While indigo itself is not found in plants, species such as *Indigofera tinctoria* L. (true indigo)^[^ ^69^ ^]^ and *Polygonum tinctorium* (Japanese and China indigo)^[^ ^70^ ^]^ contain indican **101** (indoxyl β‐D glucoside), the major precursor of indigo. Another precursor of indigo is Isatan B (indoxyl‐β‐D‐ketogluconate), a compound naturally found in the leaves of *Isatis tinctoria*.^[^ ^71^ ^]^

**Scheme 5:**
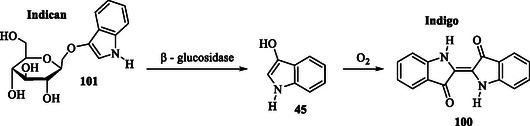
Synthesis of indigo **100**.

Natural indigo is traditionally obtained through the fermentation of leaves. During this process, indican **101** undergoes enzymatic hydrolysis, with the sugar residue cleaved by the enzyme glucosidase present in the leaves. This reaction produces indoxyl **45**, which rapidly converts into indigo **100** via oxidative dimerization (**Scheme** **5**).^[^ ^72^ ^]^ A wide range of microorganisms can produce indigo **100** through the enzymatic action of specific oxygenases and hydroxylases. This capability makes microbial fermentation a sustainable and eco‐friendly alternative for indigo synthesis.^[^ ^73^ ^]^

The formation of both intra‐ and intermolecular hydrogen bonds in indigo contributes to its elevated melting point (390–392 °C), its low solubility in most solvents, and its characteristic influence on the reflectance spectrum.^[^ ^74^ ^]^

**Figure 9:**
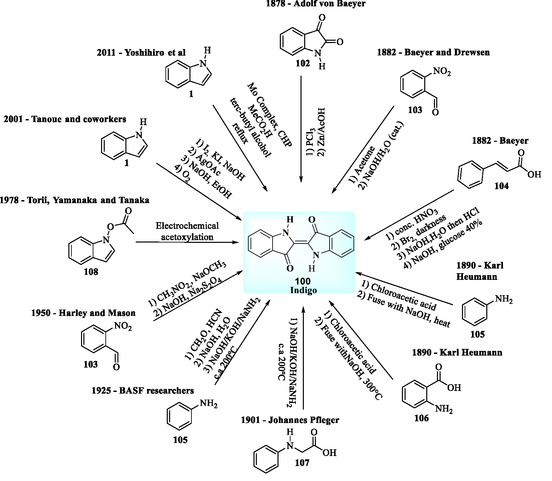
Alternative methods for the synthesis of indigo **100**.

Indigo **100**, due to its significant economic importance, has been synthesized using a variety of methods (**Figure** **9**).^[^ ^75^ ^]^ Adolf von Baeyer accomplished the synthesis of indigo starting from isatin **102** in 1878. In 1882, he developed two additional synthetic routes, one beginning with 2‐nitrobenzaldehyde **103** and the other with cinnamic acid **104**. However, these methods were not practical for large‐scale production. A few years later, in 1890, Karl Heumann achieved improved results by utilizing aniline **105** (version 1) and anthranilic acid **106** (version 2) in his process. Notably, Adolf von Baeyer was awarded the 1905 Nobel Prize in Chemistry for his pioneering work on indigo synthesis and structural elucidation.^[^ ^76^ ^]^

Figure 9 presents selected examples of alternative methods developed by various researchers for the synthesis of indigo (**100**),^[^ ^77^ ^]^ utilizing different starting materials (**1** and **102–108**).

**Scheme 6:**
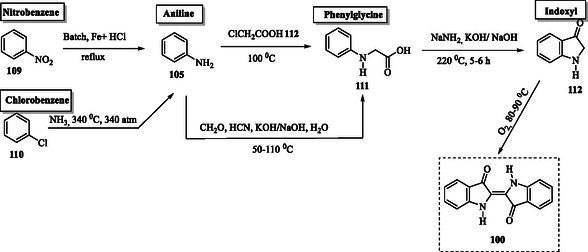
Chemical processes in indigo **100** manufacturing.

The second version of Heumann's method was eventually scaled up for industrial production by BASF (Germany). However, a more cost‐effective and practical approach to synthesizing indigo was introduced in 1901 by Johannes Pfleger, who refined Heumann's original process (version 1). BASF chemists further enhanced the synthesis of *N*‐phenylglycine by starting with the petroleum‐based compound aniline **105** (**Scheme** **6**).^[^ ^78^ ^]^ Historically, aniline **105** was synthesized from nitrobenzene **109** via a batch process, where nitrobenzene **109** was reduced using elemental iron and hydrochloric acid (HCl) under reflux conditions.^[^ ^76^ ^]^

From the 1940s until 1966, Dow developed an alternative method, producing aniline from chlorobenzene **110** and ammonia (Scheme 6). This process operated at a temperature of 340 °C and pressures of up to 340 atm. Aniline **105** reacts with chloroacetic acid at 100 °C to form *N*‐phenylglycine **111**. By the 1920s, prior to Dow's method, this route had been largely replaced by an alternative process involving the reaction of aniline with formaldehyde (CH_2_O), hydrogen cyanide (HCN), a caustic alkali (KOH or NaOH), and water. *N*‐Phenylglycine **111** was then fused to form indoxyl **112** under an inert atmosphere using sodamide (NaNH_2_) and caustic alkali (KOH/NaOH) at 220 °C for 5–6 h. The resulting indoxyl **112** was subsequently oxidized by air at 80–90 °C to produce indigo **100**. This modification not only optimized the process but also provided significant economic benefits.^[^ ^76^ ^]^

Today, various isomers and derivatives of indigo can be readily synthesized, expanding its applications beyond traditional uses.^[^ ^75^ ^]^ Indigo carmine (IC) dye, despite being classified as toxic to rats, pigs, and humans, is widely used in the textile industry.^[^ ^79^ ^]^ Tetrabromoindigo was produced through the bromination of synthetic indigo. The most commonly employed industrial method involved bromination in a nitrobenzene solvent, as indicated by publications from DuPont and BASF employees, which exclusively reference this approach.^[^ ^76^ ^]^

### 4.6 Material Science: Indoles as Corrosion Inhibitors

**Scheme 7:**
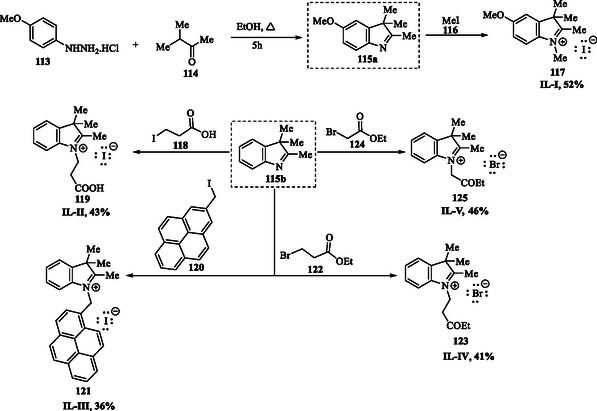
Preparation of indolium‐based ionic liquids (**117**, **119**, **121**, **123**, and **125**).

Acid solutions are widely utilized in numerous industrial processes, including acid pickling and descaling. To prevent undesired metal dissolution, organic inhibitors containing heteroatoms with strong adsorption affinity are commonly introduced into these aggressive environments. Most organic inhibitors contain heteroatoms that enhance their adsorption onto the metal surface, forming a protective barrier that shields the metal from the corrosive environment. Ahmed et al. reported the synthesis of five indolium‐based ionic liquids, 5‐methoxy‐1,2,3,3‐tetramethyl‐3*H*‐indolium iodide **117** (IBIL‐I), 1‐(2‐carboxyethyl)‐2,3,3‐trimethyl‐3*H*‐indolium iodide **119** (IBIL‐II), 2,3,3‐trimethyl‐1‐(pyren‐2‐ylmethyl)‐3*H*‐indolium iodide **121** (IBIL‐III), 1‐(3‐ethoxy‐3‐oxopropyl)‐2,3,3‐trimethyl‐3*H*‐indolium bromide **123** (IBIL‐IV), and 1‐(2‐ethoxy‐2‐oxoethyl)‐2,3,3‐trimethyl‐3*H*‐indolium bromide **125** (IBIL‐V) (**Scheme** **7**), as inhibitors for the corrosion of mild steel in acid medium, by reacting the compound **115a,b** with a variety of halides **116**, **118**, **120**, **122**, and **124** in anhydrous toluene at 80 °C.^[^ ^80^ ^]^

The adsorption behavior and corrosion inhibition efficacy of these IBILs were investigated using polarization measurements and cyclic voltammetry. The results revealed that the inhibition efficiency of mild steel corrosion in 0.5 M H_2_SO_4_ is strongly influenced by the molecular structure of the IBILs. Notably, bromide‐counterion IBILs exhibited superior inhibitory performance compared to those with iodide.^[^ ^80^ ^]^

### 4.7 Material Science: Indoles as Antifouling Agents

As the demand for sustainable solutions grows, minimizing environmental impact while maintaining industrial efficacy has become essential. In this regard, developing compounds with low toxicity to marine ecosystems and high efficiency in protecting vessels and submerged structures has gained significant importance. Within this context, Ni et al. (2021) conducted a study on the synthesis and application of indole ester derivatives containing acrylamide as antifouling agents, aiming to enhance marine biofouling prevention while ensuring environmental compatibility.^[^ ^81^ ^]^

**Scheme 8:**
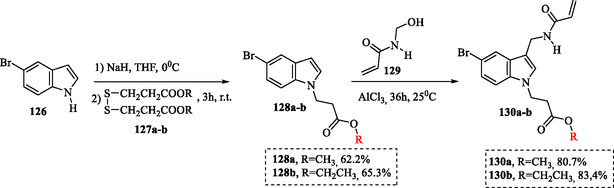
Synthesis of indole ester derivatives containing acrylamide groups as polymerization monomers.

In algal inhibition and antiprotein adsorption assays of antifouling paints, the indole derivatives exhibited strong efficacy in preventing biological adhesion, outperforming the reference compound, chlorothalonil. These results highlight their potential as environmentally friendly antifouling agents for marine protection applications. To obtain the target compounds **130a,b**, the intermediates **128a,b** were first synthesized via a Michael reaction between 5‐bromo‐1*H*‐indole **126** and dimethyl β‐dithiopropionates **127a,b** in a tetrahydrofuran (THF) medium, using NaH as a base. Subsequently, a Friedel–Crafts reaction between the derivatives **128a,b** and *N*‐methylol acrylamide **129**, catalyzed by aluminum chloride, led to the formation of the final target substances **130a,b** (**Scheme** **8**).^[^ ^81^ ^]^

### 4.8 Material Science: Indole‐Based Material with Enhanced Iodine Adsorption Capacity

**Scheme 9:**
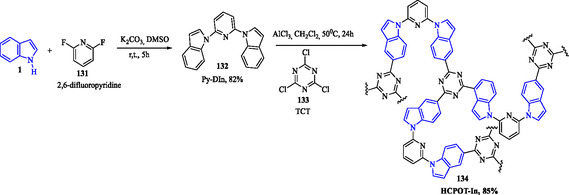
Synthesis of HCPOT‐In, **134**.

Yuan et al. (2022) investigated the synthesis and iodine adsorption capacity of a nitrogen‐rich porous organic polytriazine containing indole rings, designated as HCPOT‐In **134**. This study aimed to develop highly efficient materials for capturing environmental pollutants, particularly residual iodine from nuclear facilities. Compound **132** was obtained by reacting compound **1** with 2,6‐difluoropyridine **131** in the presence of a base at room temperature. This polymer was synthesized via a Friedel–Crafts reaction between 2,6‐di(1*H*‐indol‐1‐yl)pyridine **132** and 2,4,6‐trichloro‐1,3,5‐triazine **133**, catalyzed by a Lewis acid (**Scheme** **9**).^[^ ^82^ ^]^

Adsorption studies revealed that HCPOT‐In **134** effectively captures iodine, primarily due to its highly porous, nitrogen‐rich structure, which facilitates strong interactions with iodine molecules. Moreover, the material exhibited significant potential for CO_2_ capture, demonstrating regeneration capability after appropriate treatment, enabling its reuse in successive adsorption cycles.^[^ ^82^ ^]^

### 4.9 Material Science: Fluorescent Probe for Selective Metal Ion Detection

**Scheme 10:**
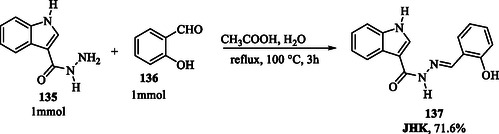
Synthesis of JHK **137** probe.

Fluorescent probes capable of detecting metal ions through emission variations serve as powerful tools in chemical analysis, offering real‐time, nondestructive detection with high sensitivity and selectivity. In this context, Li et al. (2024) conducted a study on the application of a probe called JHK (**137**; **Scheme** **10**). The organic compound was synthesized through a reaction between indole hydrazide **135** and salicylaldehyde **136** in an aqueous medium, catalyzed by acetic acid.^[^ ^83^ ^]^

The study emphasized the use of selective methods to distinguish Al^3+^ from other ions, which is crucial for preventing false‐positive results and ensuring analytical accuracy. In conducted tests, the JHK probe demonstrated excellent anti‐interference capability, maintaining fluorescence intensity even in the presence of ions that typically cause interference.^[^ ^83^ ^]^

Experiments on living organisms, such as zebrafish, demonstrated that the JHK probe (Scheme 10) effectively enables fluorescent imaging, providing researchers with a valuable tool for visualizing aluminum ions in biological systems. These findings suggest that the JHK probe holds significant potential for applications in biological and environmental studies, where precise Al^3+^ detection is crucial due to its association with neurodegenerative diseases like Alzheimer's and Parkinson's, as well as its potential adverse effects on plant growth.^[^ ^83^ ^]^

### 4.10 Material Science: Pure Blue‐Emitting Organic Compounds for Optoelectronic Applications

Pure blue‐emitting organic compounds are a class of organic light‐emitting diode (OLED) materials that generate blue light upon electrical excitation. They hold great promise for next‐generation light source technology,^[^ ^84^ ^]^ particularly in commercial displays for televisions, smartphones, tablets, and other advanced electronic devices.^[^ ^85^ ^]^ Despite significant research progress, developing pure blue OLEDs remains a challenge due to their short operational lifetime. This limitation underscores the need for ongoing research to enhance their stability and performance.^[^ ^84^ ^]^

**Figure 10:**
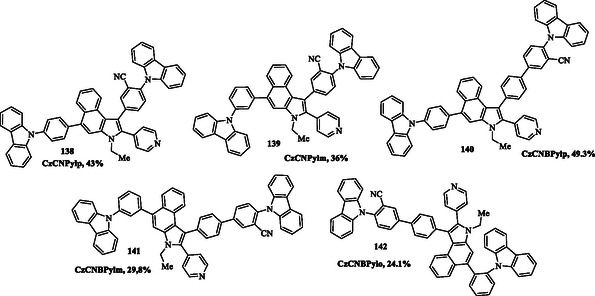
Chemical structures of blue fluorescent benzoindole‐based compounds **138–142** designed for OLED applications.

Ye et al. synthesized deep blue fluorescent materials based on a benzoindole scaffold, functionalized with a phenyl‐carbazole as an electron‐donating group and pyridine and cyano moieties as electron‐accepting groups **138–142** (**Figure** **10**). The electroluminescent properties of the synthesized materials highlight their potential for deep blue OLED applications. Among them, the CzCNBPylp **140** molecule stands out for its high luminance, low operating voltage, and excellent thermal stability. These are key factors for the long‐term performance of OLED devices, making them a promising candidate for commercial display applications.^[^ ^86^ ^]^

### 4.11 Material Science: Polyurea as Promising Material for Advanced Coating Applications

Polyurea is a promising material with applications in coatings, fibers, adhesives, and biomedical implants. Traditionally, its synthesis relies on isocyanates, which pose significant health and environmental risks. In this context, Kumari et al. reported an innovative isocyanate‐free method for synthesizing polyureas **150a–d** (R‐PUrea), integrating triazolinediones **149a–d** (R‐*bis*TAD) and indole cores **144** into the polymer backbone as a sustainable alternative to isocyanates.^[^ ^87^ ^]^

The synthesis of R‐PUrea was achieved via a highly efficient TAD‐indole click reaction, a streamlined synthetic approach in which the 1,2,4‐triazoline‐3,5‐diones **149a–d** rapidly react with indole derivative **144** under mild conditions.^[^ ^87^ ^]^

**Scheme 11:**
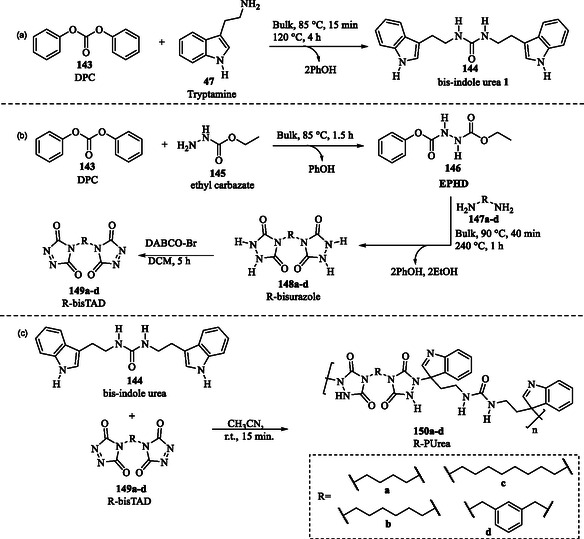
a) Synthesis of *bis*‐indole urea **144**. b) Synthesis of R‐*bis*TAD **149a–d**. c) Synthesis of polyurea **150a–d** via TAD‐indole reaction.

The synthesis involved two parallel steps. First, *bis*‐indole urea **144** was synthesized by reacting diphenyl carbonate **143** (DPC) with tryptamine **47** under catalyst‐free and solvent‐free conditions (**Scheme** **11**a). Simultaneously, DPC **143** and ethyl carbazate **145** underwent a nucleophilic substitution, generating the intermediate (**146)**, which subsequently reacted with diamines **147a–d** to afford the corresponding R‐*bis*urazoles **148a–d**. These intermediates were then oxidized, leading to the formation of R‐*bis*TAD **149a–d** (Scheme 11b). The polymerization step, involving the reaction of *bis*‐indole urea **144** with the corresponding *bis*‐TADs **149a–d**, resulted in the rapid formation of R‐PUrea polymers **150a–d** within only 15 min under mild conditions (Scheme 11c).^[^ ^87^ ^]^

The molecules **150a–d** were employed in the synthesis of highly stable hydrophobic polyurea coatings. When applied to silicon surfaces, these coatings exhibited excellent hydrophobicity. Furthermore, the incorporation of indole‐derived moieties into their backbone conferred strong photoluminescence properties, making them highly promising for optical applications.^[^ ^87^ ^]^

The diverse applications of indole compounds across various sectors have fueled extensive research on this nucleus in multiple fields. Its versatility in organic synthesis, particularly in expanding structural diversity, underscores its significance. While numerous review articles have explored the biological aspects of indoles,^[^ ^1^, ^5^, ^59^, ^88^, ^89^, ^90^, ^91^, ^92^, ^93^, ^94^, ^95^, ^96^ ^]^ and others have focused on synthetic strategies for constructing the indole scaffold,^[^ ^8^, ^97^, ^98^, ^99^, ^100^, ^101^, ^102^, ^103^ ^]^ the present work provides a comprehensive overview of the applications and synthetic methodologies developed over the past five years for the construction and functionalization of the indole nucleus.

## 5 Pioneering and Classical Methods for Indole Synthesis: From Baeyer's Discovery to the Classical Synthetic Approaches

**Scheme 12:**
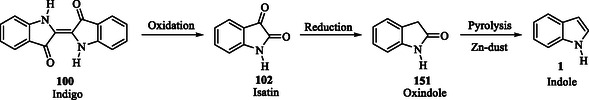
Baeyer's method for indole **1** synthesis.

The term “indole” is derived from India, where it was first isolated from the blue dye indigo **100** in the 16th century. In 1886, Adolf Baeyer successfully isolated indole through the pyrolysis of oxindole **151** with zinc dust (**Scheme** **12**). Oxindole **151** itself was initially synthesized by reducing isatin **102**, which is produced through the oxidation of indigo **100**. Today, indole **1** is primarily synthesized from coal tar, making it one of the most widely distributed heterocyclic compounds. It forms the core structure of thousands of naturally occurring alkaloids, pharmaceutical drugs, and other bioactive molecules.^[^ ^104^ ^]^

**Scheme 13:**
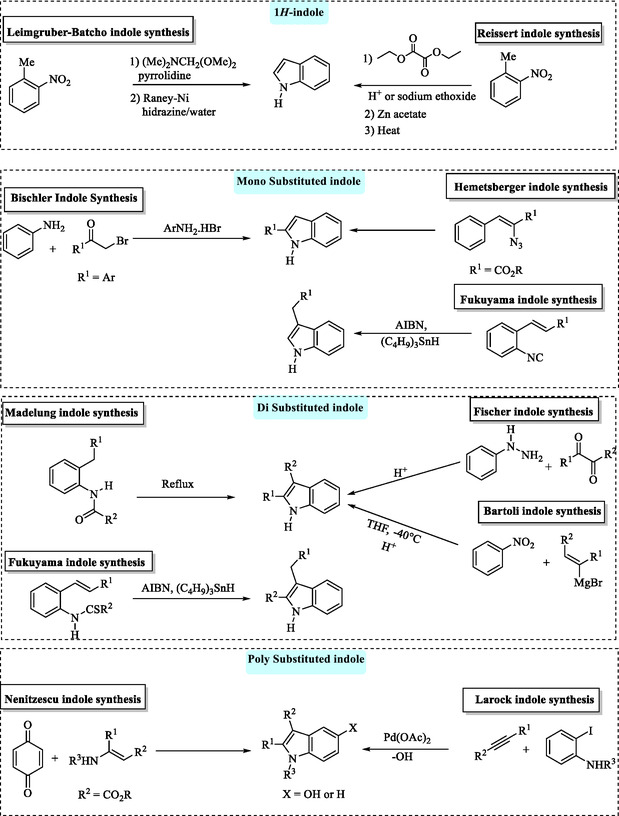
Classical methods for indole synthesis.

Since Baeyer's first indole synthesis, more than 20 named reactions have been developed,^[^ ^105^ ^]^ including the Fischer indole synthesis,^[^ ^106^, ^107^, ^108^ ^]^ Madelung synthesis,^[^ ^109^ ^]^ Reissert synthesis,^[^ ^110^ ^]^ Bartoli indole synthesis,^[^ ^111^, ^112^ ^]^ Nenitzescu synthesis,^[^ ^113^ ^]^ Bischler indole synthesis,^[^ ^114^, ^115^, ^116^ ^]^ Hemetsberger indole synthesis,^[^ ^117^ ^]^ Larock indole synthesis,^[^ ^118^, ^119^, ^120^, ^121^ ^]^ Fukuyama indole synthesis,^[^ ^122^, ^123^ ^]^ and Leimgruber–Batcho indole^[^ ^124^, ^125^ ^]^ have been developed (**Scheme** **13**).

Bugaenko et al. provided a review on modified versions of classical synthetic methods, including the Fischer synthesis, Nenitzescu synthesis, Ullmann reaction, Leimgruber–Batcho synthesis, Reissert synthesis, Bartoli reaction, Madelung synthesis, and Cadogan–Sundberg reaction (Scheme 13).^[^ ^97^ ^]^

## 6 Synthesis of Indoles via Direct Functionalization of Indole Derivatives: Metal‐Free Approaches

### 6.1 Boron‐Mediated Directed Aromatic C—H Hydroxylation Under Metal‐Free Conditions

**Scheme 14:**
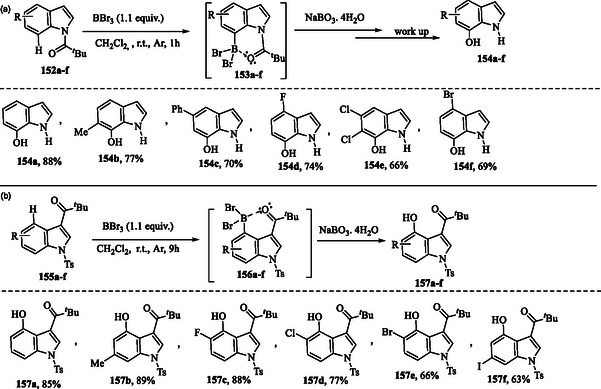
Metal‐free hydroxylation via C—H borylation of a) *N*‐pivaloyl indoles **152a–f** and b) *N*‐tosyl indoles **155a–f** using BBr_3_.

The strategy involves the mild, directed C—H borylation of *N*‐pivaloyl (*N*‐Piv) indoles **152a–f** using BBr_3_ under metal‐free conditions. This process leads to the formation of dibromoborane species **153a–f**, which can then be oxidized with NaBO_3_. The protocol demonstrates excellent functional group tolerance, and the *N*‐Piv group can be spontaneously removed during the work‐up with K_2_CO_3_, yielding the N—H free C7‐hydroxylated indoles **154a–f** (**Scheme** **14**a).^[^ ^126^, ^127^ ^]^

The employment of C3‐substituted pivaloyl indoles **155a–f** with BBr_3_ in DCM solvent results in the formation of C4‐dibromoborane species **156a–f**. These intermediates can then be efficiently converted into hydroxylation products **157a–f** in a one‐pot process using NaBO_3_·4H_2_O (Scheme 14b).^[^ ^126^, ^127^ ^]^

**Scheme 15:**
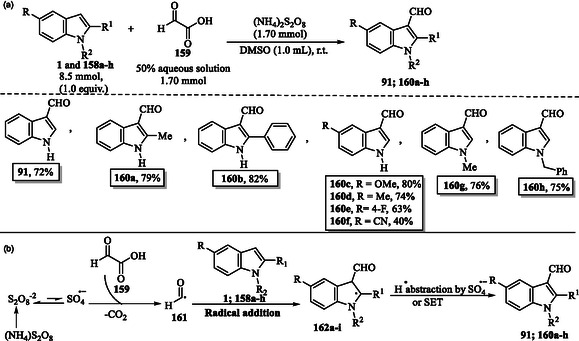
a) Metal‐free decarboxylative formylation of indoles **91**–**160a–h** using oxo‐ketoacid and (NH_4_)_2_S_2_O_8_ and b) plausible mechanism.

A metal‐free approach for the formylation of indoles **1** and **158a–h** using ammonium persulfate (NH_4_)_2_S_2_O_8_ via direct decarboxylative cross‐coupling of compound **161** is presented, achieving **91** and **160a–h** with moderate to good yields (**Scheme** **15**).^[^ ^128^, ^129^ ^]^

In the plausible mechanism, the sulfate radical anion generated from ammonium persulfate abstracts a hydrogen radical from available α‐keto acid **159**, leading to the formation of formyl radical **161** with the release of CO_2_. The resulting radical species then adds to indoles **1** and **158a–h**, forming intermediates **162a–i**. These intermediates subsequently undergo hydrogen atom abstraction by a sulfate radical anion or a single‐electron transfer (SET) process, ultimately yielding the corresponding carbonyl products **91** and **160a–h**.^[^ ^128^, ^129^ ^]^

Other formylation reactions,^[^ ^129^, ^130^, ^131^ ^]^ such as the visible‐light‐mediated C3 formylation of indole catalyzed by Eosin Y (EY), which employs tetramethylethylenediamine (TMEDA) as the carbon source and air as the oxidant,^[^ ^132^ ^]^ have also been reported in the literature.

### 6.2 Sulfuryl Chlorofluoride‐Mediated Controlled Chlorination and Chlorooxidation of Unprotected Indoles

**Scheme 16:**
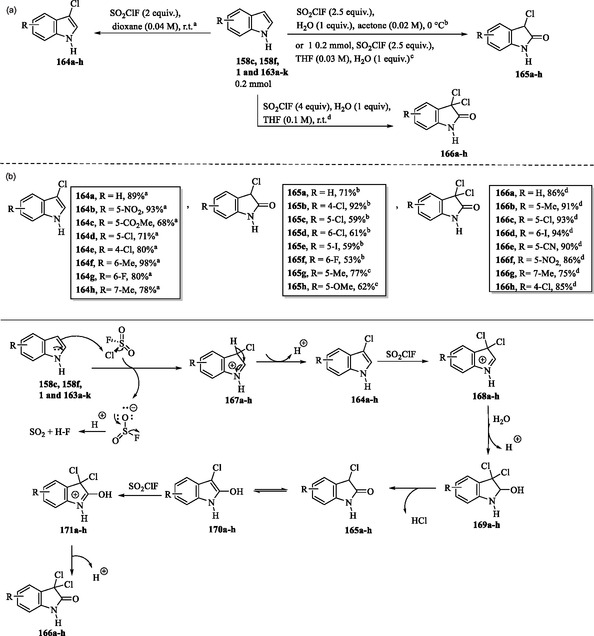
a) Chlorination and chlorooxidation of unprotected indoles **1**, **158c**, **158f**, and **163a–k** and b) proposed mechanism.

Sulfuryl chlorofluoride has proven to be a versatile reagent for the selective chlorination and chlorooxidation of simple, unprotected indoles **1**, **158c**, **158f**, and **162a–k**. By simply switching the reaction solvents, three distinct products, 3‐chloroindoles **164a–h**, 3‐chloro‐2‐oxindoles **165a–h**, and 3,3‐dichloro‐2‐oxindoles **166a–h**, can be selectively obtained in good to excellent yields (**Scheme** **16**a).^[^ ^133^ ^]^

In the proposed mechanism, SO_2_ClF, due to the high electronegativity of fluorine, possesses an electrophilic chlorine atom that allows the electrophilic chlorination of indole derivatives **1**, **158c**, **158f**, and **163a–k**, yielding iminium ions **167a–h**. In this reaction, SO_2_ClF acts as a source of chlorine cations. Concurrently, the resulting sulfuryl fluoride anion reacts with H^+^ to release gaseous SO_2_ and HF. Subsequent deprotonation of **167a–h** yields the key 3‐chloroindoles **164a–h**. A further attack on **164a–h** by a second chlorine cation produces iminium ions **168a–h**, which are then converted into the hemiaminal intermediates **169a–h**. Elimination of HCl from dichloride compounds **169a–h** generates the corresponding monochloride derivatives **165a–h**, which can subsequently undergo tautomerization to yield hydroxyindoles **170a–h**. These compounds are then subjected to further chlorination to form intermediates **171a–h**, followed by deprotonation to afford the final products **166a–h** (Scheme 16b).^[^ ^133^ ^]^

### 6.3 Quaternary Ammonium Salts as Efficient Alkylating Agents in Organic Synthesis

**Scheme 17:**
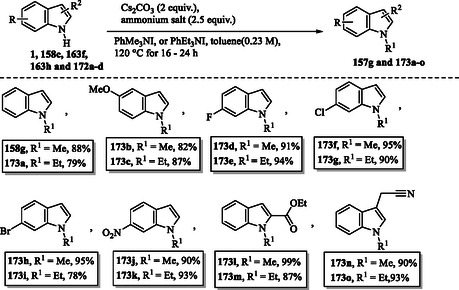
*N*‐Methylation and *N*‐ethylation of indoles using quaternary ammonium salts as alkylating agents.

Monoselective *N*‐methylation and *N*‐ethylation of indole structures **1**, **158c**, **163f**, **163h**, and **172a–d** using solid, nontoxic, and easy‐to‐handle quaternary ammonium salts as methylating and ethylating agents under mildly basic conditions were reported by Templ et al. The resulting methylated and ethylated indoles **158g** and **173a–o** were obtained in good yields (**Scheme** **17**).^[^ ^134^, ^135^ ^]^

### 6.4 Eco‐Friendly Halogenation of Indoles Using Oxone‐Halide System

**Scheme 18:**
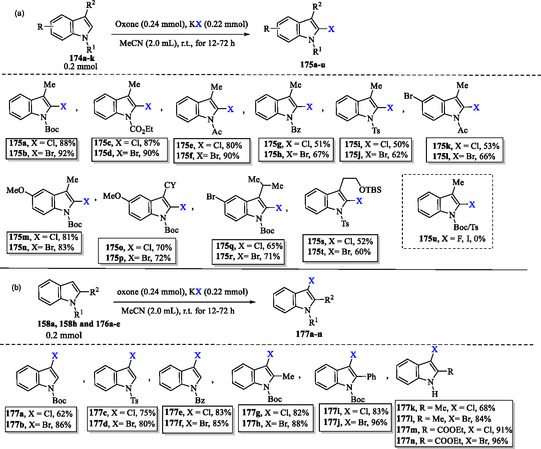
Eco‐friendly halogenation of indoles using the Oxone‐halide system a) at position C2 and b) at position C3.

In **Scheme** **18**a, the presence of electron‐withdrawing groups, such as carbamate, acetyl, benzoyl, or sulfonate, on the nitrogen atom of indoles facilitates mild C2 chlorination and bromination of compounds **174a–k**. This transformation is achieved using stoichiometric halide salts in the presence of Oxone, leading to the formation of the corresponding halogenated products **175a–u**. It was noted that the corresponding fluoriation and iodination using this protocol (KF/Oxone or KI/Oxone) did not work.^[^ ^136^ ^]^

In contrast, selective C3 chlorination and bromination of compounds **158a**, **158h**, and **176a–e** with stoichiometric halide and Oxone can lead to products **177a–n**, independent of the electronic properties of the protecting group on the indole nitrogen (Scheme 18b).^[^ ^136^ ^]^

## 7 Synthesis of Indoles via Direct Functionalization of Indole Derivatives: Metal‐Catalyzed Approaches

### 7.1 Palladium‐Catalyzed, Iodine‐Assisted Carbonylation of Indoles Using ClCF_2_CO_2_Na and Alcohols

**Scheme 19:**
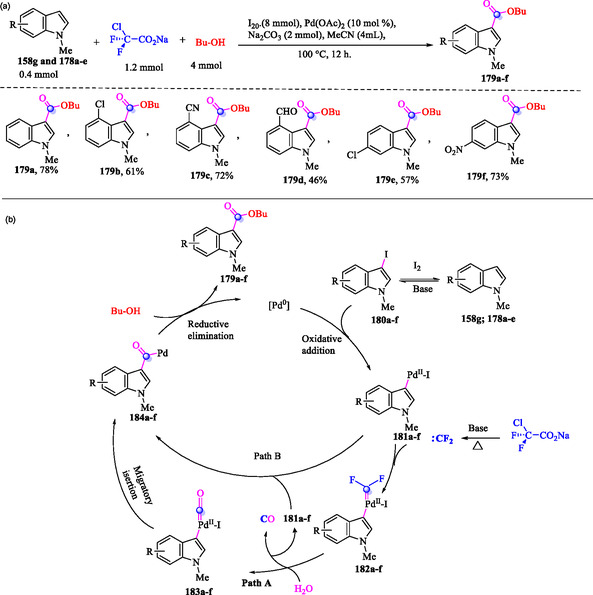
a) Palladium‐catalyzed, iodine‐assisted carbonylation of indoles **158g** and **178a–e** using ClCF_2_CO_2_Na and butanol and b) plausible mechanism.

Cao et al. have reported a palladium‐catalyzed, iodine‐assisted carbonylation of indoles **158g** and **178a–e** using ClCF_2_CO_2_Na and butanol, providing a practical and efficient strategy for regioselective functionalization of indoles (**Scheme** **19**a). According to the proposed mechanism, **158g** and **178a–e** undergo a regioselective reaction with I_2_ in the presence of a base to afford 3‐iodo‐1‐methylindole derivatives **179a–f**.

Next, the Pd^0^ species undergoes oxidative addition with **180a–f** to form Pd(II) complexes **181a–f**, which trap the in situ generated difluorocarbene, providing the [Pd(II)–CF_2_] intermediates **182a–f** (Path A, Scheme 19b). Subsequently, hydrolysis of intermediates **182a–f** generates Pd complexes **183a–f**, which then undergo migratory insertion to form the acyl‐Pd(II) species **184a–f**. Alternatively, the [Pd(II)–CF_2_] intermediates **182a–f** could undergo hydrolysis, regenerating **181a–f** and releasing CO. These regenerated species would then be converted into the acyl‐Pd(II) intermediates **184a–f** (Path B, Scheme 19b). Finally, alcohol insertion, followed by reductive elimination, furnishes the target products **179a–f** while simultaneously regenerating the Pd^0^ species. The authors reported that when 20 equivalents of H_2_^18^O were used under otherwise identical conditions, gas chromatography‐mass spectrometry analysis detected 40% incorporation of ^18^O into product **179a**. This study confirmed that the carbonyl oxygen atom in product **179a** originated from water present in the reaction system.^[^ ^137^ ^]^

### 7.2 Transition Metal‐Mediated C—H Activation of Indole

**Scheme 20:**
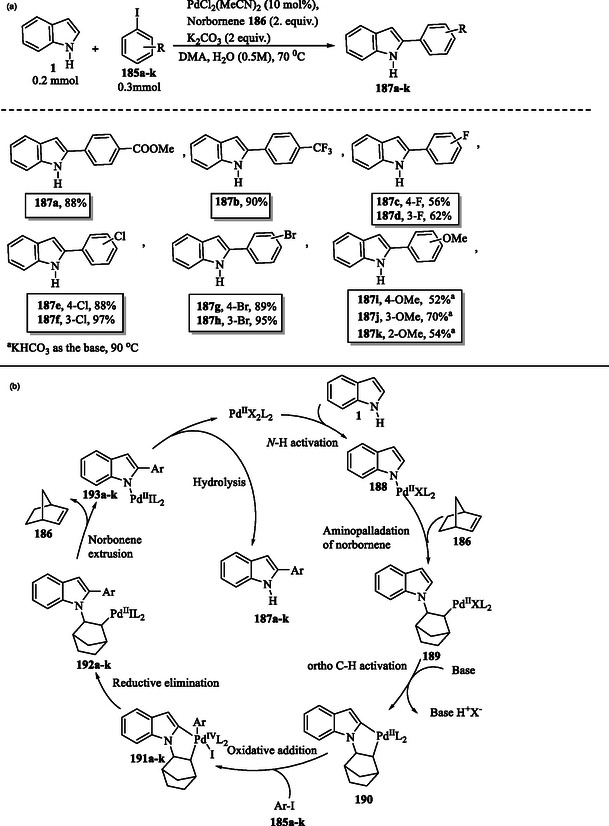
a) Arylation of indole ring and b) plausible mechanism.

A synthetically valuable strategy to enable C—H activation of indoles involves the use of transition metal catalysts. The application of catalysts such as palladium, rhodium, iridium, ruthenium, and manganese has transformed organic synthesis, facilitating selective alkynylation, arylation, acylation, and annulation reactions.^[^ ^127^, ^138^, ^139^ ^]^ One example is the palladium‐catalyzed direct C2‐arylation reaction of free (N–H) indoles that was developed utilizing a norbornene‐mediated C—H activation process to yield compounds **187a–k** (**Scheme** **20**a).^[^ ^140^ ^]^

The catalytic cycle for palladium‐catalyzed direct C2 arylation begins with the *N*‐palladation of indole **1**, where Pd(II) coordinates to its nitrogen atom to form the palladium–indole complex **188**. The coordinated indole undergoes aminopalladation with norbornene **186**, leading to the formation of the Pd(II) intermediate **189**. *Ortho* C—H activation at the C2 position of the indole ring in intermediate **189** results in the formation of the palladacyclic species **190**. The oxidative addition of aryl iodides **185a–k** leads to the formation of Pd(IV) intermediates **191a–k**. This step is followed by a reductive elimination, leading to the formation of 2‐aryl‐*N*‐palladaindoles **192a–k**, which subsequently undergo norbornene extrusion to yield intermediates **193a–k**. Subsequent hydrolysis of **193a–k** releases the final 2‐arylindole products **187a–k** and regenerates the Pd(II) catalyst (Scheme 20b).^[^ ^140^ ^]^

### 7.3 Palladium‐Medium Dearomatization‐Rearomatization for N‐Alkylation of Indoles

**Scheme 21:**
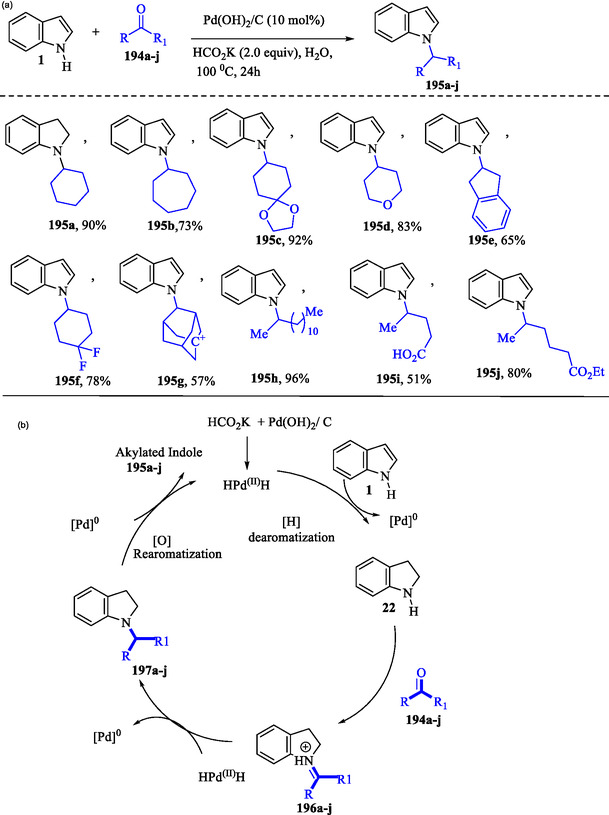
a) Synthesis of *N‐*alkylated indoles **195a–j** and b) proposed mechanism.

Wang et al. reported the synthesis of *N‐*alkylated indoles **195a–j** using ketones **194a–j** as alkylating agents through a dearomatization–rearomatization strategy. This strategy facilitates the reductive cross‐coupling of indole **1** with ketones **194a–j** under aqueous conditions (**Scheme** **21**a). In the plausible mechanism (Scheme 21b), the HPd^II^H species is generated by reacting Pd(OH)_2_/C with potassium formate and water. The reduction of indole **1** by HPd^II^H induces dearomatization of the pyrrole ring, generating indoline **22**, which subsequently condenses with ketones **194a–j** to form the iminium intermediates **196a–j**. The latter then undergoes further reduction to produce *N*‐alkylated indolines **197a–j**. Finally, indolines **197a–j** are converted via oxidative rearomatization to produce *N*‐alkylated indoles **195a–j**, concurrently regenerating HPd^II^H.^[^ ^141^ ^]^

### 7.4 Synthesis of Indole via Metal‐Catalyzed Dehydrogenation of Indolines

**Scheme 22:**
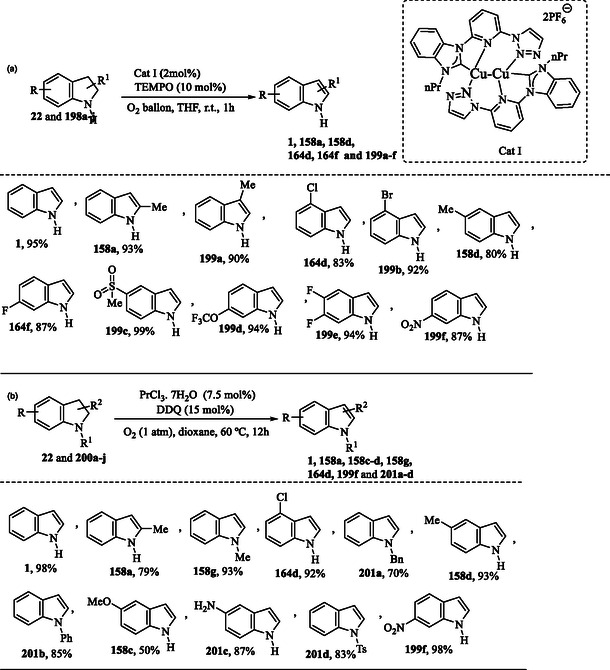
Preparation of indole by dehydrogenation of indoline a) Copper‐catalyzed and b) praseodymium‐catalyzed.

A copper‐catalyzed dehydrogenation reaction enables the synthesis of indoles **1, 158a, 158 d, 164d, 164f,** and **199a–f** (**Scheme** **22**a) from various indolines **22** and **198a–j**, using molecular oxygen (O_2_) as a green oxidant. The oxidation proceeds efficiently at room temperature in THF, with 10 mol% TEMPO employed as an additive to facilitate the reaction.^[^ ^142^ ^]^

In Scheme 22b, a praseodymium‐catalyzed aerobic dehydrogenative aromatization of indolines **22** and **200a–j** under mild conditions allowed the synthesis of indole derivatives **1**, **158a**, **158c–d**, **158g**, **164d**, **199f**, and **201a–f** in good yields.^[^ ^143^ ^]^

Additional examples of reactions involving indoline dehydrogenation have been reported in the literature.^[^ ^144^, ^145^ ^]^

### 7.5 Metal‐Catalyzed C3‐ or N‐Alkylation of Indolines

**Scheme 23:**
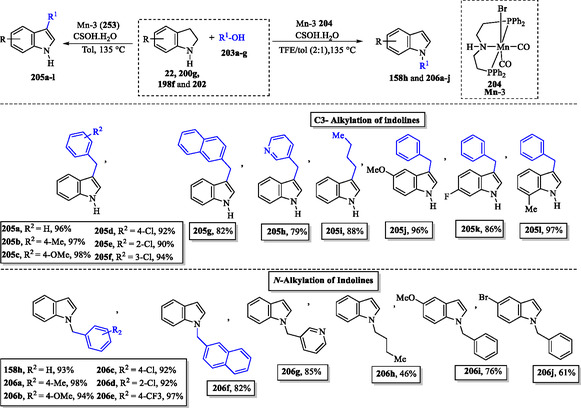
Transition‐metal‐mediated catalytic alkylation at the C3 or N1 position of indoles.

A regioselective dehydrogenative alkylation of indolines **22**, **200g**, **198f**, and **202** is achieved using readily available alcohols **203a–g** as alkylating agents. This transformation employs a single air‐ and moisture‐stable manganese catalyst **204** and allows selective access to either C3‐ or *N*‐alkylated indoles, depending on the solvent used. This regioselective methodology produced C‐alkylated **205a–l** and *N‐*alkylated **158h** and **206a–j** indoles in good to excellent yields (**Scheme** **23**a,b).^[^ ^146^ ^]^

The regioselective C—H and N—H bond functionalization of indolines using alcohols in water, mediated by an iridium catalyst, is well documented in the literature.^[^ ^147^ ^]^

## 8 Metal‐Free Methods for Construction of Indole Derivatives

### 8.1 Synthesis of Indole from 2‐Alkenylanilines

**Scheme 24:**
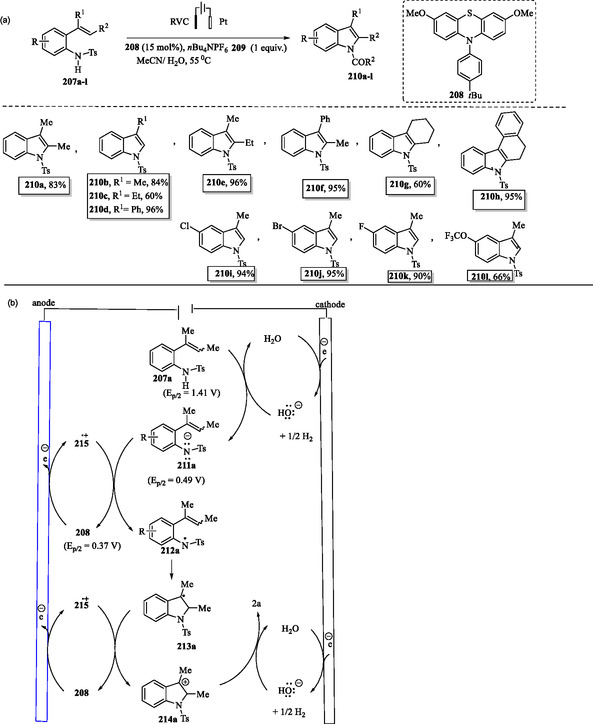
a) Electrocatalytic synthesis of indoles **210a–l** and b) plausible mechanism.

An electrocatalytic approach for indole synthesis via dehydrogenative cyclization of 2‐vinylanilides **207a–l** was described by Zheng et al. This reaction employs an organic redox catalyst **208** and proceeds without the need for an external oxidant, enabling the rapid and efficient synthesis of 3‐substituted and 2,3‐disubstituted indoles **210a–l** (**Scheme** **24**a).^[^ ^148^ ^]^

In the plausible mechanism (Scheme 24b), the electrochemical process begins with the anodic oxidation of catalyst **208** to its radical cation **215**·^+^, accompanied by the cathodic reduction of solvent H_2_O, producing H_2_ and hydroxide ions (HO^−^). The hydroxide ion HO^−^ generated at the cathode deprotonates tosylamine **207a** [pKa (TsNHPh) = 8.46 in H_2_O], forming its conjugate base **211a**. Subsequent cyclization of intermediate **212a** furnishes compound **213a**, which undergoes further oxidation by **215**·^+^ to yield **214a**. Deprotonation of **214a** then affords the final indole product **210a**.^[^ ^148^ ^]^

Other methods for transforming 2‐vinyl anilines into indoles have been described in the literature.^[^ ^149^, ^150^, ^151^, ^152^, ^153^, ^154^, ^155^, ^156^, ^157^ ^]^

### 8.2 Desulfonylative Chlorocyclization of N,N‐Disubstituted 2‐Alkynylanilines for the Synthesis of 3‐Chloroindoles

**Scheme 25:**
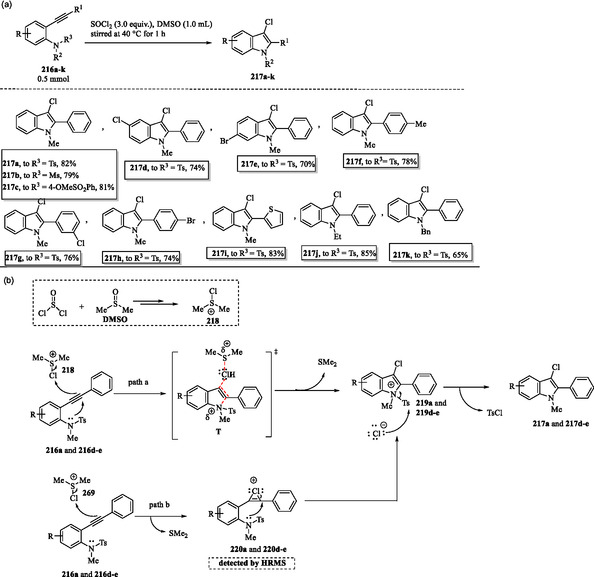
a) DMSO/SOCl_2_‐Mediated synthesis of 3‐chloroindoles **217a–k** and b) plausible mechanism.

The DMSO/SOCl_2_ system promoted the intramolecular cyclization and chlorination of *N,N*‐disubstituted 2‐alkynyl anilines **216a–k**, affording a series of 3‐chloroindoles **217a–k** in moderate to good yields (**Scheme** **25**a). In the mechanism, Li et al. suggested that DMSO initially reacts with the electrophilic SOCl_2_, forming a reactive dimethyl chloride sulfonium intermediate **218** via an interrupted Pummerer reaction. Given that the chlorine atom is incorporated into the indole framework, it is proposed that *N*‐tosylated 2‐alkynylanilines **216a** and **216d–e** follow a concerted pathway, in which they attack the chlorine atom of the reactive sulfonium intermediate **218** through transition state **T**, resulting in the formation of key cyclized intermediates **219a** and **219d–e**. Finally, the tosyl (Ts) group is eliminated via nucleophilic attack by a free chloride ion, affording the 3‐chloroindoles **217a–k** (Scheme 25b, path a).^[^ ^158^ ^]^

The authors suggest that an alternative mechanistic pathway, potentially involving the formation of chloronium ion intermediates **220a** and **220d–e**, cannot be excluded. The reaction of alkynes **216a** and **216d–e** with the reactive dimethylchlorosulfonium intermediate **218** generates chloronium ions **220a** and **220d–e**, which then undergo intramolecular cyclization to form the corresponding intermediates **219a** and **219d–e**. Finally, 3‐chloroindoles **217a** and **217d–e** are obtained from intermediates **219a** and **219d–e** through chloride ion‐mediated elimination of the tosyl (Ts) group (Scheme 25b, path b). Notably, high‐resolution mass spectrometry analysis provides supporting evidence for the formation of the chloronium ion intermediates **220a** and **220d–e**.^[^ ^158^ ^]^

### 8.3 Metal‐Free PPA‐Mediated Fischer Indole Synthesis via Tandem Hydroamination‐Cyclization of Simple Alkynes with Arylhydrazines

**Scheme 26:**
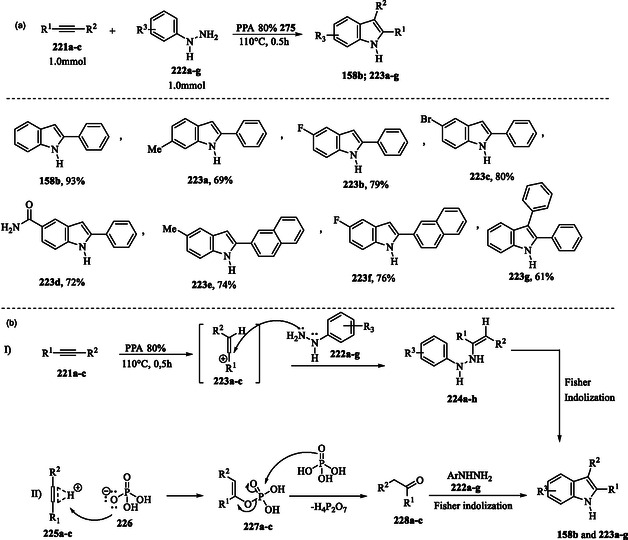
a) Bronsted acid‐catalyzed hydrohydrazination of alkynes **221a–c** with arylhydrazines **222a–g** and b) plausible mechanisms.

In a recent study, Aksenov and coworkers reported a modified Fischer indole synthesis that employs polyphosphoric acid (PPA) as a catalyst to facilitate the hydroamination of alkynes **221a–c** with arylhydrazines **222a–g**. This approach provides an efficient and metal‐free strategy for the synthesis of the indole compounds **158b** and **223a–g**, thereby enhancing the synthetic versatility of the Fischer indolization process. The protocol proposed by the authors presents a promising alternative to the conventional metal‐catalyzed method, which typically depends on the use of excess zinc. The synthetic method using excess PPA (P_2_O_5_, 80% w/w) as both catalyst and solvent proved highly efficient, yielding indoles **158b** and **223a–g** in good to excellent yields at 110 °C within just 30 min (**Scheme** **26**a).^[^ ^159^ ^]^

The authors proposed the mechanism through two possible pathways. In the first pathway (Scheme 26b–I), protonation of alkynes **221a–c** by PPA generates vinyl cations **223a–c**, which undergo direct nucleophilic attack by hydrazines **222a–g** to form ene‐hydrazines **224a–h**. These compounds are subsequently transformed through a classical Fischer indole synthesis.^[^ ^159^ ^]^ The second possible mechanism (Scheme 26b–II) involves a nucleophilic attack by the conjugate base of phosphoric acid **226** on the protonated alkyne, resulting in the formation of vinyl phosphate intermediates **227a–c**. Under anhydrous conditions, vinyl phosphate derivatives **227a–c** undergo acidolysis, a chemical reaction in which a molecule is cleaved by an acid, to produce the corresponding ketones **228a–c**. The final step involves a nucleophilic attack by phenylhydrazines **222a–g** on the carbonyl group of compounds **228a–c**, followed by Fischer indolization.^[^ ^159^ ^]^

### 8.4 Electrochemical Synthesis of Substituted Indoles from 1‐(2‐Aminophenyl)ethanols

**Scheme 27:**
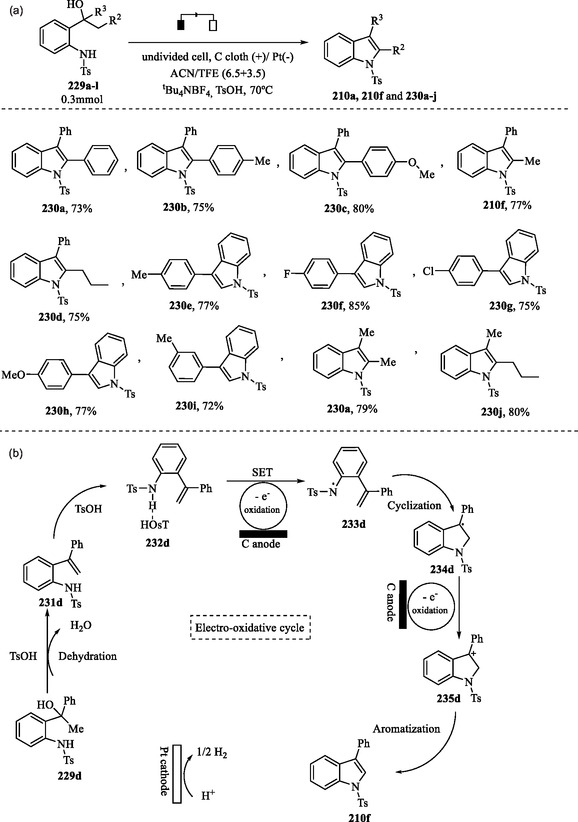
a) Electrosynthesis of substituted indoles **210a**, **210f**, and **230a–j** and b) proposed mechanism.

Yuan et al. proposed a green and straightforward electrochemical methodology for the synthesis of substituted indoles **210a**, **210f**, and **230a–j** from 1‐(2‐aminophenyl)alcohols **229a–l** (**Scheme** **27**a). This methodology presents several advantages over conventional methods, including the absence of metals, iodine, and stoichiometric oxidants. Additionally, it features practical reaction conditions and demonstrates tolerance to both water and air.^[^ ^160^ ^]^

The reaction is conducted in an undivided electrochemical cell equipped with a carbon cloth anode and a platinum cathode. It employs a solvent mixture of acetonitrile (ACN) and 2,2,2‐trifluoroethanol (TFE) in the presence of *p*‐toluenesulfonic acid (TsOH) and the electrolyte tetrabutylammonium tetrafluoroborate (Bu_4_NBF_4_). This protocol afforded high yields even on a gram scale. However, substituting TsOH or Bu_4_NBF_4_ led to a decline in yield, a result consistent with the mechanism proposed by the authors (Scheme 27a).^[^ ^160^ ^]^

In the proposed mechanism (Scheme 27b), dehydration of compound **229d** in the presence of TsOH leads to the formation of intermediate **231d**, which subsequently forms a hydrogen‐bonded complex **232d** with TsOH. This complex undergoes a concerted proton‐coupled electron transfer (PCET), in which single‐electron oxidation leads to the formation of the *N*‐centered radical **233d**. Cyclization of species **233d** results in the formation of the C‐centered radical **234d**, which is subsequently oxidized to the C‐centered cation intermediate **235d**. This intermediate undergoes a deprotonation reaction, leading to the formation of the indole derivative **210f**. The reduction of H^+^ at the cathode results in the formation of H_2_ gas. The authors performed mechanistic investigations employing radical trapping experiments, intermediate characterization, cyclic voltammetry, and EPR spectroscopy.^[^ ^160^ ^]^

### 8.5 HFIP‐Catalyzed Microwave‐Assisted Synthesis of Indoles

**Scheme 28:**
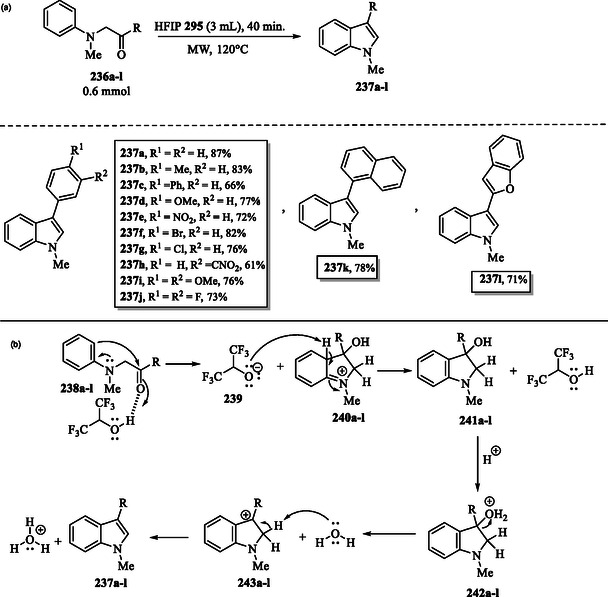
a) Microwave‐Assisted indole synthesis catalyzed by HFIP and b) proposed mechanism.

Yao et al. reported an efficient and sustainable synthesis of indole derivatives **237a–l** from substituted α‐amino arylacetones **236a–l** via the Bischler indole synthesis, using 1,1,1,3,3,3‐hexafluoropropan‐2‐ol (HFIP) as a catalyst under microwave irradiation (**Scheme** **28**a). This metal‐ and additive‐free reaction produces water as the sole byproduct and allows for easy recovery of HFIP via rotary distillation, highlighting its efficiency and sustainability as a synthetic approach.^[^ ^161^ ^]^

A plausible mechanism for the formation of compounds **237a–l** is depicted in Scheme 28b. HFIP activates the carbonyl group of α‐arylamino acetones **238a–l**, enhancing their electrophilicity and promoting Friedel–Crafts cyclization, which leads to the formation of intermediates **240a–l**. The alkoxide form of HFIP **239** abstracts a proton from intermediates **240a–l**, restoring the aromaticity of the benzene ring and leading to the formation of the corresponding indolinols **241a–l**. Subsequently, the hydroxyl group in indolinols **241a–l** is protonated, forming intermediates **242a–l**. Dehydration of intermediates **242a–l** generates carbocation species **243a–l**, which subsequently undergo deprotonation to yield the desired final products **237a–l**.^[^ ^161^ ^]^

### 8.6 LiN(SiMe_3_)_2_/CsF‐Mediated Tandem Madelung Indole Synthesis

**Scheme 29:**
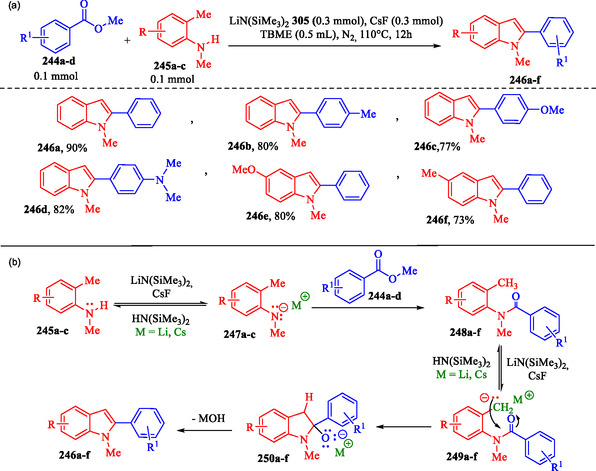
a) Madelung tandem indole synthesis and b) mechanism pathway.

Mao et al. developed a synthetic method for N‐methyl‐2‐phenylindoles **246a–f** via a modified Madelung Tandem indole synthesis, utilizing benzoates **244a–d**, *N*‐methyl‐*o*‐toluidines **245a–c**, lithium *bis*(trimethylsilyl)amide LiN(SiMe_3_)_2_, and cesium fluoride (CsF) (**Scheme** **29**a). The combination of LiN(SiMe_3_)_2_ and CsF forms a mixed‐base system, identified as the key factor responsible for the high efficiency of the reaction.^[^ ^162^ ^]^ The authors proposed a mechanism (Scheme 29b) that begins with the mixed‐base system [LiN(SiMe_3_)_2_/CsF], which preferentially deprotonates the nitrogen atom of *N*‐methyl‐*o*‐toluidines **245a–c**, generating intermediates **247a–c**. This facilitates a nucleophilic addition to the carbonyl group of methyl benzoates **244a–d** via acyl substitution, leading to the formation of amide derivatives **248a–f**. Subsequently, the benzylic C—H bond of amides **248a–f** is reversibly deprotonated by the mixed‐base system. The resulting benzylic anions **249a–f** undergo intramolecular cyclization via nucleophilic attack on the carbonyl group, forming intermediates **250a–f** and ultimately yielding the desired N‐methyl‐2‐phenylindoles **246a–f**.^[^ ^162^ ^]^

### 8.7 Metal‐Free Intramolecular Cyclization of N‐Acyl Amides for the Synthesis of 2‐(Per)Fluoroalkyl‐3‐Nitroindoles

**Scheme 30:**
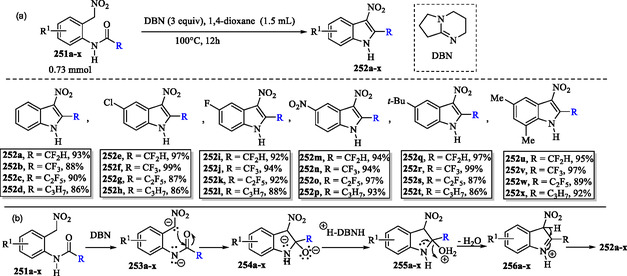
a) Metal‐free synthesis of 2‐(per)fluoroalkyl‐3‐nitro indoles **251a–x** and b) plausible mechanism.

Sterligov and coworkers developed a metal‐free protocol for the efficient synthesis of 3‐nitro‐2‐(*per*)fluoroalkyl indoles **252a–x** in excellent yields via intramolecular cyclization of *N*‐acyl amides **251a–x** (**Scheme** **30**a).^[^ ^163^ ^]^

In the proposed mechanism (Scheme 30b), deprotonation of benzyl compounds **251a–x** by 1,5‐diazabicyclo[4.3.0]non‐5‐ene (DBN, **313**) generates carbanion intermediates **253a–x**. These carbanions subsequently undergo intramolecular nucleophilic attack on the amide carbonyl group, resulting in the formation of cyclized species **254a–x**. The subsequent steps involve nitrogen protonation and double protonation of the oxygen atom, facilitated by the conjugated acid of DBN, leading to the formation of species **255a–x**. Subsequently, the nitrogen lone pair facilitates the elimination of H_2_O. Finally, indoles **252a–x** are formed via proton elimination from species **256a–x**, a step also promoted by the conjugated acid of DBN.^[^ ^163^ ^]^

### 8.8 Metal‐Free Visible‐Light‐Induced Synthesis of Indoles Using an Organophotoredox Catalyst

**Scheme 31:**
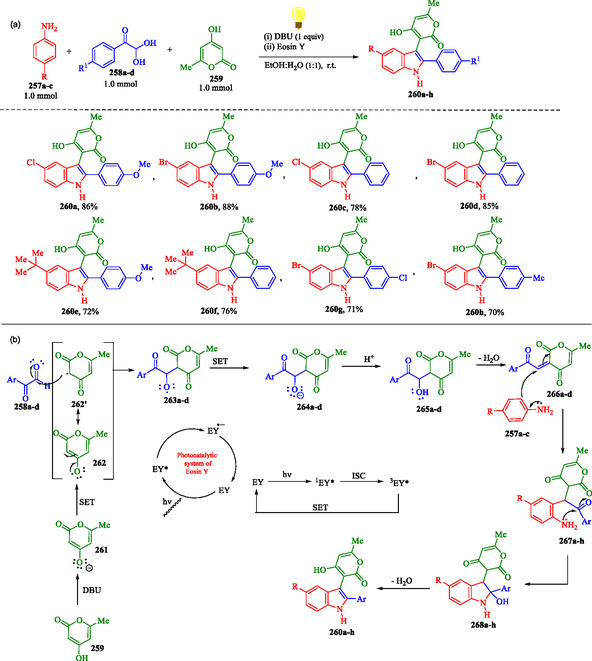
a) Eco‐friendly synthesis of 3‐functionalized indole derivatives **260a–h** and b) proposed mechanism.

Yadav et al. reported an eco‐friendly three‐component reaction involving substituted anilines **257a–c**, 2,2‐dihydroxy‐1‐(phenyl)ethan‐1‐ones **258a–d**, and 4‐hydroxy‐6‐methyl‐2*H*‐pyran‐2‐one **259** (**Scheme** **31**a).^[^ ^164^ ^]^

This transformation utilizes EY as an organophotoredox catalyst, providing a sustainable and metal‐free alternative to conventional inorganic transition metal catalysts. This protocol operates under mild conditions, delivers high atom economy, and ensures a clean reaction profile by using an EtOH:H_2_O solvent system and atmospheric oxygen as the oxidant. Upon absorbing visible light, the organophotoredox catalyst EY is excited to its singlet state (^1^EY*), which then undergoes intersystem crossing (ISC) to the more stable triplet state (^3^EY*). In this triplet state, it participates in a SET process. DBU abstracts a proton from 4‐hydroxy‐6‐methyl‐2*H*‐pyran‐2‐one **259**, generating anion **261**.^[^ ^164^ ^]^

The photocatalytic cycle is initiated by the excitation of EY upon irradiation with visible light. Subsequently, a SET occurs from anion **261** to the excited state of EY. This electron transfer generates radical **262**, which, through resonance, forms species **262′** that subsequently traps phenylglyoxals **258a–d** (α‐ketoaldehydes), leading to the formation of intermediates **263a–d**. At this stage, EY returns to its ground state via oxidative quenching, transferring an electron to intermediates **263a–d**, and generating the corresponding species **264a–d**. Protonation of species **264a–d** yields intermediates **265a–d**, which subsequently undergo dehydration to afford compounds **266a–d**. A subsequent nucleophilic attack by aromatic amines **257a–c** on derivatives **266a–d** leads to the formation of **267a–d**, which undergo intramolecular cyclization, generating the intermediate **334a–d**, which undergoes dehydration, ultimately yielding the final products **260a–h**.^[^ ^164^ ^]^

### 8.9 Mechanochemical Fischer Indole Synthesis

**Scheme 32:**
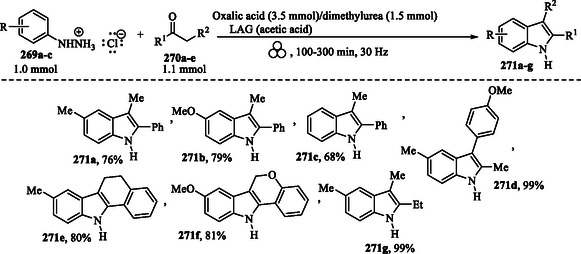
Mechanochemical Fischer indoles **271a–g** synthesis.

In 2022, D’Auria et al. reported a sustainable, solvent‐free mechanochemical method for the synthesis of Fischer indoles (**Scheme** **32**). By grinding phenylhydrazines **269a–c**, carbonyl compounds **270a–e**, oxalic acid, dimethylurea (DMU), and acetic acid in a ZrO_2_ milling jar, the authors efficiently synthesized indoles **271a–g** under solvent‐free mechanochemical conditions. The results are consistent with those of the traditional Fischer indole synthesis, with reaction outcomes influenced by the steric and electronic properties of the substituents.^[^ ^165^ ^]^

### 8.10 PIFA‐Promoted Oxidative Cyclization of Electron‐Rich α‐Arylhydrazones

**Scheme 33:**
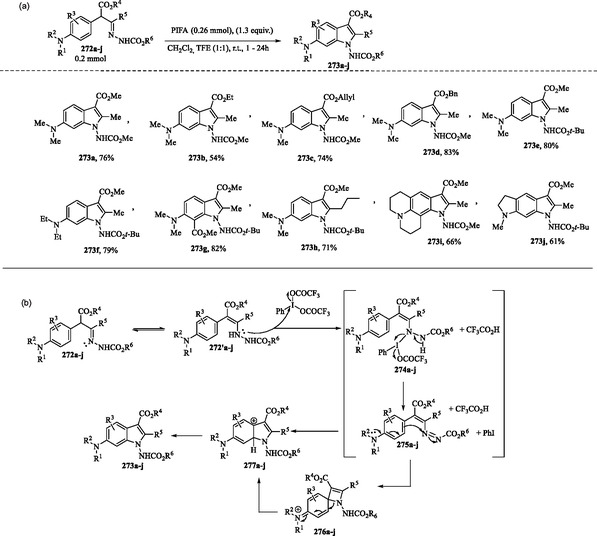
a) Intramolecular oxidative C—N bond formation for the synthesis of substituted 1‐aminoindoles **273a–j** from electron‐rich α‐arylhydrazones **272a–j** and b) proposed mechanism.

Yadava and coworkers recently reported a metal‐free oxidative cyclization of electron‐rich α‐arylhydrazones promoted by phenyliodine *bis*(trifluoroacetate) (PIFA), to afford *N*‐amino‐1*H*‐indoles **273a–j** in good yields (**Scheme** **33**a).^[^ ^166^ ^]^

A proposed mechanism for the PIFA‐mediated cycloamination is depicted in Scheme 33b. Following the initial CH/NH tautomerization (1,3‐H shift), the oxidation of ene‐hydrazines **272′a–j** by PIFA is proposed to generate the reactive azo compounds **274a–j**. This sequence involves the nucleophilic attack of **272′a–j** on the electrophilic iodine center of PIFA, resulting in the formation of intermediates **274a–j** with the concurrent loss of a CF_3_CO_2_H molecule. Subsequently, simultaneous release of PhI and CF_3_CO_2_H from **274a–j** generates the transient azoalkene species **275a–j**.^[^ ^166^ ^]^

Starting from **275a–j**, an intramolecular nucleophilic attack of the aromatic ring on the azo group takes place, followed by rearomatization of the cationic intermediates **277a–j**, ultimately leading to the formation of the desired *N*‐amino‐1*H*‐indoles **273a–j**. The authors also propose that the intramolecular cyclization of **277a–j** can proceed via an *ipso* attack, followed by rearrangement (via N‐to‐C bond migration) of the cationic spiro dieniminium salts **276a–j** as intermediates.^[^ ^166^ ^]^

### 8.11 Multicomponent Synthesis of Tetrazolo‐Indoles

**Scheme 34:**
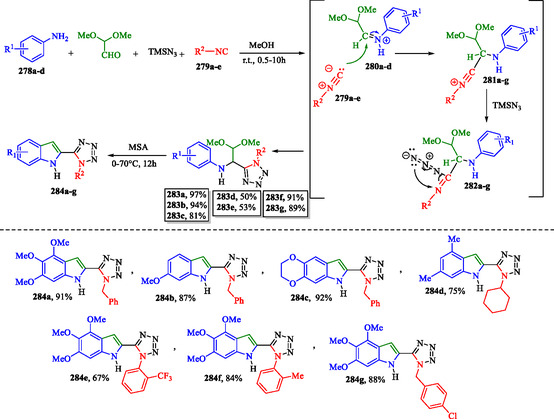
Synthesis of tetrazole‐indole **284a–g** derivatives via the Ugi‐tetrazole reaction.

The Ugi–Tetrazole reaction is a valuable variation of the classic Ugi multicomponent reaction (MCR), specifically designed to incorporate a tetrazole ring into the final product. In the classical Ugi reaction, an aldehyde or ketone, an amine, an isocyanide, and a carboxylic acid react to form an amide. In the Ugi‐Tetrazole variation, the carboxylic acid is replaced by an azide source (such as NaN_3_), leading to the formation of a tetrazole ring, a five‐membered nitrogen‐containing structure with four nitrogen atoms. In **Scheme** **34**, the reaction begins with the condensation of aromatic amines **278a–d** with dimethoxyacetaldehyde, resulting in the formation of Schiff bases **280a–d**.^[^ ^167^ ^]^ These amino derivatives are subsequently converted into nitrilium ions **281a–g** upon the addition of substituted isocyanides **279a–e**. (TMSN_3_) attacks species **281a–g**, leading to the formation of intermediates **282a–g**, which then undergo an irreversible sigmatropic rearrangement to yield the tetrazole products **283a–g**.^[^ ^168^ ^]^

The tetrazole derivatives **283a–g** undergo cyclization to indole compounds under acidic conditions, furnishing products **284a–g**. Anhydrous methanesulfonic acid **357** (MSA) provided the highest cyclization efficiency. In contrast, higher water content promotes side reactions, thereby reducing the overall yield.^[^ ^167^ ^]^

### 8.12 Sustainable Multicomponent Synthesis of Indole‐2‐Carboxamides

**Scheme 35:**
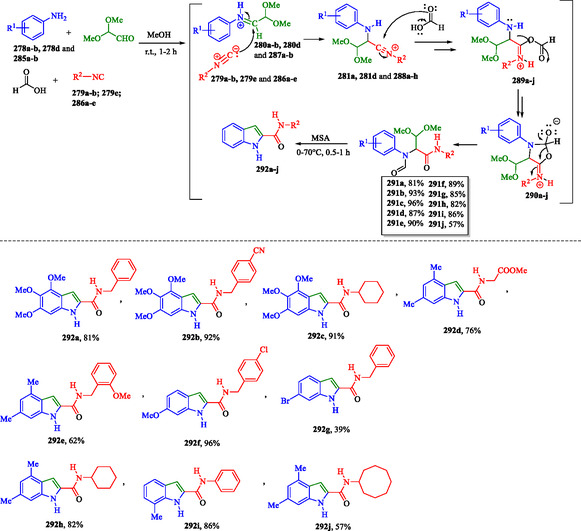
Synthesis of indole‐2‐carboxamide derivatives **292a–j** via MCR.

Lei et al. proposed another alternative metal‐free strategy for the synthesis of indoles via MCR. In this method, a series of indole‐2‐carboxamides **292a–j** were efficiently synthesized in good yields through a one‐pot, two‐step procedure. The process involves a four‐component Ugi reaction (U‐4CR) with aromatic amines **278a,b**, **278d**, and **285a,b**, glyoxal dimethyl acetal, formic acid, and substituted isocyanides **279a,b**, **279e**, and **286a–e**, followed by cyclization induced by MSA (**Scheme** **35**).^[^ ^169^ ^]^

Initially, the condensation of aromatic amines **278a,b**, **278d**, and **285a,b** with glyoxal dimethyl acetal forms iminium intermediates **280a,b**, **280d**, and **287a,b**, which subsequently react with substituted isocyanides **279a,b**, **279e**, and **286a–e** to generate nitrilium ion species **281a**, **281d**, and **288a–h**. These intermediates undergo nucleophilic addition by formic acid, leading to the formation of protonated imidates **289a–j**. Subsequently, intramolecular nucleophilic addition on intermediates **289a–j** leads to the formation of cyclic compounds **290a–j**, which then undergo intramolecular rearrangement to yield the Ugi adducts **291a–j**. Finally, the MSA‐induced cyclization of **291a–j** (Scheme 35) affords the desired indole‐2‐carboxamides **292a–j**.^[^ ^169^, ^170^, ^171^ ^]^

## 9 Synthesis of Indole Derivatives via Metal‐Catalyzed Reactions

### 9.1 Synthesis of Indole Derivatives via Anilines Generated from Cyclohexanones Using the Pd/C–Ethylene System

**Scheme 36:**
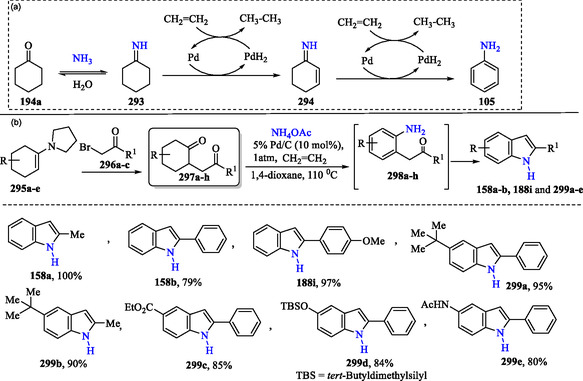
a) Plausible mechanism for Pd/C−ethylene mediated synthesis of aniline **105** from cyclohexanone **194a** and ammonium acetate and b) application of this method in the synthesis of indole.

Maeda et al. reported the direct synthesis of aniline **105** from cyclohexanone **194a** and ammonium acetate under nonaerobic conditions, employing a Pd/C–ethylene system. In **Scheme** **36**a, cyclohexanone **194a** reacts with ammonia to form cyclohexanimine **293**, which sequentially undergoes two Pd/C‐catalyzed hydrogen transfer steps with ethylene, yielding cyclohex‐2‐en‐1‐imine **294** and ultimately aniline **105**. Using this efficient synthetic protocol, a diverse range of substituted indoles **158a–b**, **188i**, and **299a–e** were obtained in high to excellent yields (Scheme 36b) from aromatic amines **298a–h**. These aromatic amines were generated in situ through the reaction of dicarbonyl compounds **297a–h** with ammonium acetate under the catalytic influence of a Pd/C–ethylene system. The derivatives **297a–h** were obtained via the reaction of α‐brominated ketones **296a–c** with 1‐(cyclohex‐1‐en‐1‐yl)pyrrolidine derivatives **295a–e**.^[^ ^105^ ^]^

### 9.2 Microwave‐Assisted Synthesis of 3‐(Trifluoromethyl)Indoles via Cerium(IV)‐Catalyzed Oxidative Cyclization

**Scheme 37:**
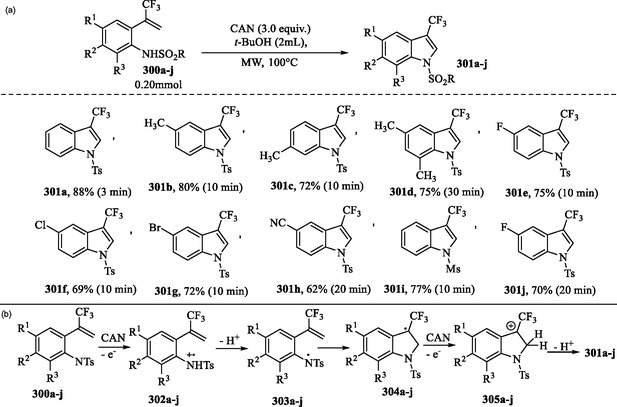
a) Microwave‐assisted synthesis of 3‐(trifluoromethyl)indoles **301a–j** via oxidative cyclization with CAN and b) proposed mechanism.

The 3‐(trifluoromethyl)indole derivatives **301a–j** were efficiently synthesized in high yields and short reaction times via microwave‐assisted intramolecular oxidative cyclization of *o*‐sulfonamido‐α‐(trifluoromethyl)styrenes **300a–j**, catalyzed by cerium(IV) ammonium nitrate (CAN) in *tert*‐butyl alcohol, as illustrated in **Scheme** **37**a.^[^ ^172^ ^]^

The proposed mechanistic pathway for the oxidative cyclization of *o*‐sulfonamido‐α‐(trifluoromethyl)styrenes **300a–j** is illustrated in Scheme 37b. Initially, CAN promotes the oxidation of the nitrogen atom in compounds **300a–j**, generating the corresponding aminium radical species **302a–j**. These radicals are subsequently deprotonated to afford the aminyl radicals **303a–j**. The radical‐initiated 5‐endo‐trig cyclization then proceeds via intramolecular nucleophilic addition of the aminyl radical to the alkene moiety in intermediates **303a–j**, resulting in the formation of the cyclized species **304a–j**. This step is followed by a one‐electron oxidation of intermediates **304a–j** with CAN, and subsequent deprotonation of the resulting carbocation species **305a–j**, affording 3‐(trifluoromethyl)indoles **301a–j**.^[^ ^172^ ^]^

### 9.3 Palladium‐Catalyzed Chlorocyclization of Unmasked 2‐Alkynylanilines for the Synthesis of 3‐Chloroindoles

**Scheme 38:**
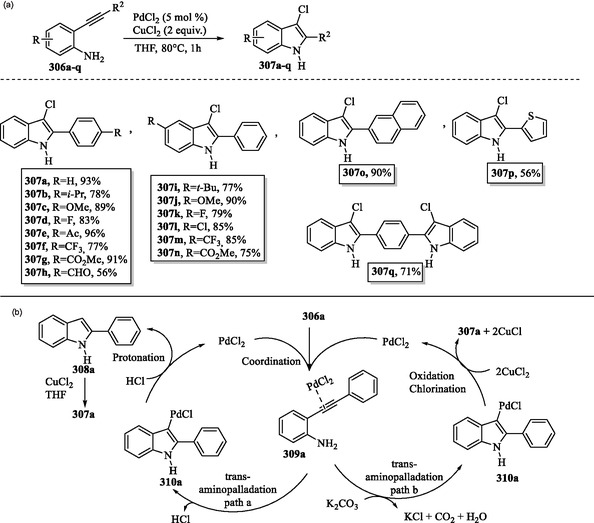
a) Palladium‐catalyzed chlorocyclization of 2‐alkynylanilines **306a–q** for the synthesis of 3‐chloroindoles **307a–q** and b) proposed mechanism.

Zheng et al. developed an efficient and practical one‐pot method for the synthesis of 3‐chloroindoles **307a–q**, achieving moderate to excellent yields via a palladium‐catalyzed chlorocyclization of 2‐alkynylanilines **306a–q**. This approach tolerates a wide variety of functional groups (**Scheme** **38**a).^[^ ^173^ ^]^

The proposed mechanism begins with the coordination of PdCl_2_ to 2‐alkynylaniline **306a**, forming the intermediate **309a**. From this point, two possible reaction pathways can proceed. In the absence of a base (Scheme 38b–path a), intermolecular transaminopalladation generates intermediate **310a**, accompanied by the release of HCl. Subsequent protonolysis of intermediate **308a** by HCl promotes the formation of indole **307a** and simultaneously regenerates the active PdCl_2_ catalyst, thus sustaining the catalytic cycle. The oxidation and chlorination of indole **310a** with CuCl_2_ in THF yield the desired 3‐chloroindole **307a** (Scheme 38b–path b). In the presence of a base, the in situ generated HCl from intermolecular transaminopalladation is neutralized.^[^ ^173^ ^]^

### 9.4 Enantioselective Palladium‐Catalyzed Cacchi Reaction for the Synthesis of Indoles with a Chiral C2‐Aryl Axis

**Scheme 39:**
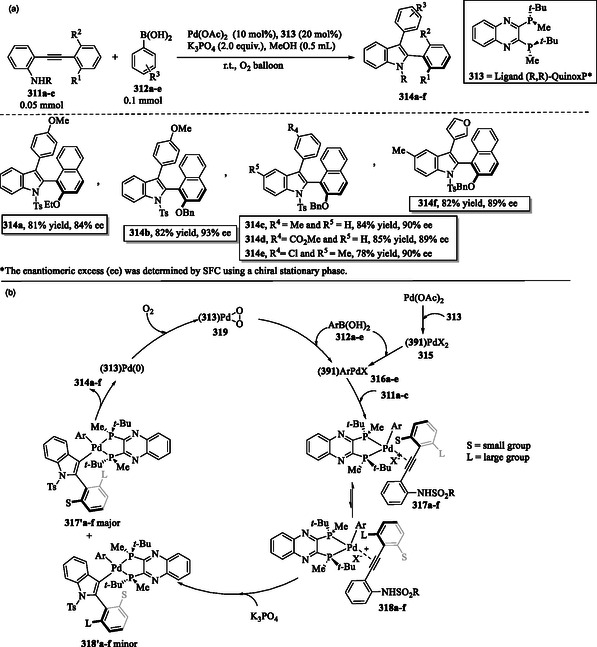
a) Enantioselective oxidative Cacchi indole synthesis and b) proposed mechanism.

He et al. reported an asymmetric oxidative Cacchi reaction between *N*‐tosyl‐2‐alkynyl anilides **311a–c** and arylboronic acids **312a–e**, enabling the efficient synthesis of axially chiral C2‐substituted indoles in high yields and with excellent enantioselectivity (**Scheme** **39**a). The reaction is carried out using Pd(OAc)_2_ and the chiral ligand (*R*,*R*)‐QuinoxP* 313 in a 2:1 ratio, with K_3_PO_4_ as the base and methanol as the solvent. This methodology was successfully applied to a broad range of substrates, demonstrating its versatility and efficiency. For instance, arylboronic acids bearing either electron‐withdrawing or electron‐donating substituents at different positions on the aromatic ring efficiently underwent arylative indolization, affording axially chiral 2,3‐disubstituted indoles **314a–f** in good to high yields with excellent enantioselectivities. The reaction predominantly afforded products with the *R* configuration, as confirmed by X‐ray diffraction analysis.^[^ ^174^ ^]^

The mechanism proposed (Scheme 39b) by the authors begins with the formation of a chiral Pd(II) complex **315**, which undergoes transmetalation with the arylboronic acids **312a–e**, generating the (**313**)ArPdX species **316a–e**. These intermediates then coordinate to the triple bond of *N‐*sulfonyl‐2‐alkynylanilides **311a–c**, forming π‐alkyne‐palladium complexes **317a–f**. This step is crucial for the formation of the asymmetric center, as the aryl substituent in complexes **317a–f** can twist out of the plane, leading to the generation of a chiral C(sp)—C(sp^2^) axis. The complexes **317a–f** are more stable than **318a–f** because the steric clash between the aryl group substituent and the QuinoxP* ligand is minimized in the former. The antiaminopalladation of substrates **317a–f** and **318a–f** predominantly affords the corresponding intermediates **317′a–f** as the major products, along with **318′a–f** as minor byproducts. Subsequent reductive elimination of **317′a–f** furnishes the enantiomerically enriched indole derivatives **314a–f** while simultaneously regenerating the Pd(0) species.^[^ ^174^ ^]^ The enantiomeric excess (ee) was determined by supercritical fluid chromatography (SFC) using a chiral stationary phase. The oxidation of Pd(0) by O_2_ forms the Pd(II) peroxo complex **319**, which rapidly reacts with arylboronic acids **312a–e** to generate palladium complexes **316a–c**, thereby completing the catalytic cycle.^[^ ^174^ ^]^

### 9.5 Synthesis of Indole Derivatives via Pd‐Catalyzed Cascade Cyclization of Alkyne‐Tethered Aryl Iodides and Diaziridinone

**Scheme 40:**
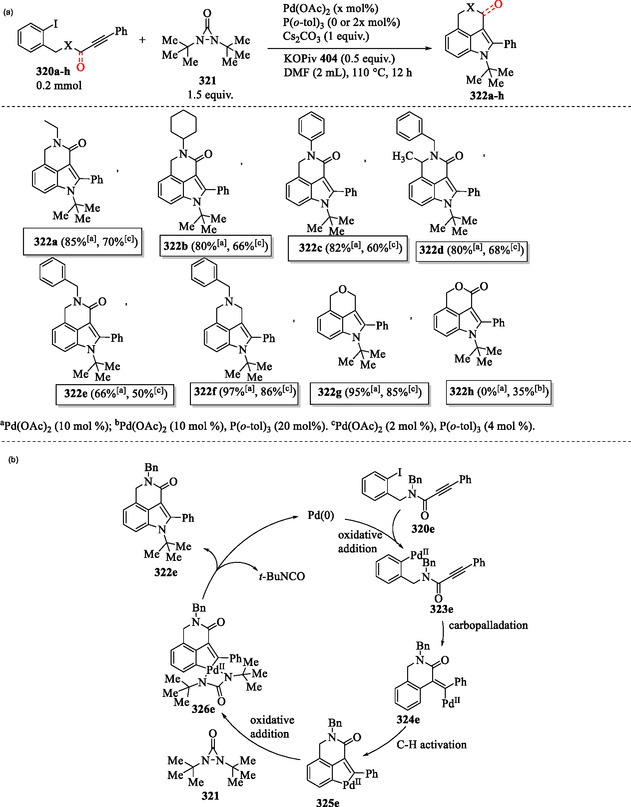
a) Synthesis of 3,4‐fused tricyclic indoles **322a–h** from Pd‐catalyzed cyclization of alkyne‐tethered aryl iodides **320a–h** with diaziridinone **321** and b) proposed mechanism.

Cheng et al. reported a Pd‐catalyzed cascade cyclization of alkyne‐tethered aryl iodides **320a–h** with diaziridinone **321**, enabling the synthesis of 3,4‐fused tricyclic indoles **322a–h**, which are key scaffolds found in various bioactive natural products and pharmaceuticals (**Scheme** **40**a). The protocol is compatible with alkyne‐tethered aryl iodides bearing both electron‐withdrawing and electron‐donating substituents. Various linkers, including amides, amines, and ethers, are well tolerated under the reaction conditions, whereas esters tend to show reduced efficiency.^[^ ^175^ ^]^

In the proposed mechanism, compound **320e** undergoes oxidative addition to Pd(0), forming the aryl‐Pd(II) complex **323e**. This intermediate then undergoes intramolecular carbopalladation, resulting in the formation of the vinyl‐Pd(II) complex **324e**. The final intermediate cleaves the aryl C—H bond, generating the C,C‐palladacycle **325e**, which then undergoes oxidative addition with *N*,*N*‐*di*‐*tert*‐butyldiaziridinone to form the pallada(IV)cycle **326e**. The latter intermediate then experiences reductive elimination to form the tricyclic indole **322e**, concurrently releasing *tert*‐butyl isocyanate (*t*BuNCO) and regenerating Pd(0) to complete the catalytic cycle (Scheme 40b).^[^ ^175^ ^]^

### 9.6 Modified Larock Indole Synthesis Using DMF‐Stabilized Palladium Clusters

**Scheme 41:**
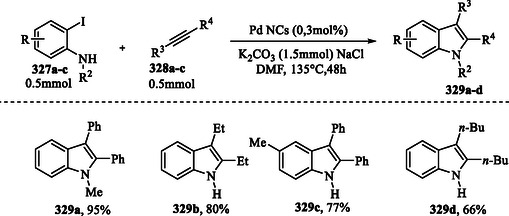
Synthesis of 2,3‐disubstituted indoles **329a–d** via a modified Larock indole approach.

Onishi et al. synthesized 2,3‐disubstituted indoles **329a–d** via the Larock indole method by reacting *o*‐iodoanilines **327a–c** with alkenes **328a‐c,** employing Pd nanocatalysts (NCs) stabilized in DMF (**Scheme** **41**). This phosphine‐free approach afforded excellent to moderate yields, particularly for substrates bearing electron‐donating groups. These catalysts offer significant advantages, including the ability to be reused up to three times and their effectiveness in very small quantities.^[^ ^176^ ^]^

### 9.7 Palladium‐Catalyzed Tandem Nucleophilic Addition and C—H Functionalization of Anilines with Bromoalkynes: An Approach to 2‐Phenylindole Synthesis

**Scheme 42:**
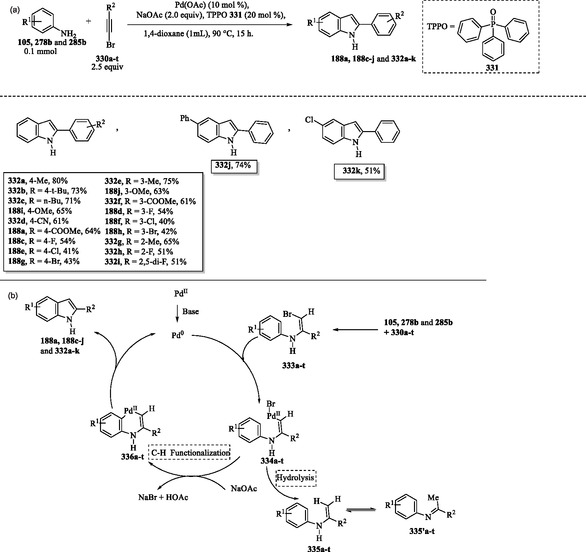
a) Indole synthesis via palladium‐catalyzed nucleophilic addition of anilines **105**, **278b** and **285b** to bromoalkynes **330a–t** and b) plausible mechanism.

A palladium‐catalyzed annulation of anilines **105**, **278b**, and **285b** with bromoalkynes **330a–t** has been developed for the synthesis of 2‐phenylindoles (**Scheme** **42**a). Scheme 42b illustrates a plausible mechanism for the formation of 2‐phenylindole derivatives **188a**, **188c–j**, and **332a–k** from anilines **105**, **278b**, and **285b** and bromoalkynes **330a–t**. The process begins with the antinucleophilic addition of anilines **105**, **278b**, and **285b** to the bromoalkynes **330a–t**, yielding (*Z*)‐*N*‐(2‐bromo‐1‐phenyl‐vinyl) anilines **333a–t**. These latter compounds then undergo oxidative addition with Pd^0^ to form species **334a–t**. Next, *ortho*‐C(sp^2^)—H functionalization of species **334a–t** results in the formation of six‐membered palladacyclic intermediates **336a–t**. Concurrently, a hydrolysis process converts the palladium species **334a–t** into *N*‐(1‐phenyl‐vinyl)anilines **335a–t**, which subsequently undergo tautomerization to afford imines **335′a–t** as side products. Finally, reductive elimination from intermediates **336a–t** affords the coupling products **188a**; **188c–j;** and **332a–k**, while simultaneously regenerating the active Pd^0^ species for the next catalytic cycle.^[^ ^177^ ^]^

Other reactions involving a catalyzed coupling between amino compounds and alkynes for the synthesis of indole derivatives have also been reported in the literature.^[^ ^178^ ^]^

### 9.8 Palladium‐Catalyzed Reductive Cyclization of β‐Nitrostyrenes Using Phenyl Formate as a CO Surrogate for Indole Synthesis

**Scheme 43:**
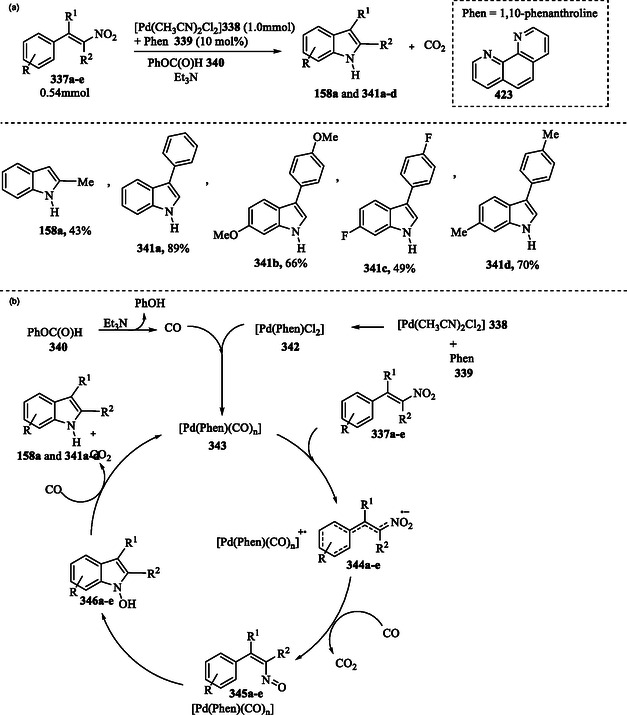
a) Palladium‐catalyzed reductive cyclization of β‐nitrostyrenes **337a–e** using phenyl formate **340** for synthesis of indole **158a** and **341a–d** and b) proposed mechanism.

In 2022, Ferreti et al. reported a palladium‐catalyzed reductive cyclization of β‐nitrostyrenes **337a–e** using phenyl formate **340** as a carbon monoxide (CO) surrogate, enabling the synthesis of indole derivatives **158a** and **341a–d** in moderate to good yields (**Scheme** **43**a). This protocol demonstrated efficiency, particularly for nitrostyrenes bearing an aryl group at the α‐position. In the absence of phenyl formate, the reaction afforded lower yields, likely due to competing oligomerization and polymerization processes promoted by the base. The presence of electron‐donating or electron‐withdrawing groups on compounds **337a–e** had minimal impact on the overall yield, with the notable exception of the dimethylamino group, which completely inhibited the reaction.^[^ ^179^ ^]^

In the mechanism proposed (Scheme 43b),^[^ ^179^ ^]^ the active catalyst **342** is initially formed by coordination of phenanthroline **339** to *bis*(acetonitrile)dichloropalladium(II) **338**. Subsequently, the reduction of palladium(II) **342** to palladium(0) species **343** occurs via CO release, generated from the decomposition of phenyl formate **340**.

The resulting complex **343** then facilitates the activation of nitrostyrenes **337a–e** via an electron transfer process. Based on general orbital considerations, the authors proposed the formation of radical anions **344a–e**. However, they also referenced literature reports demonstrating that the trans–cis isomerization of β‐nitrostyrenes has been experimentally observed following the formation of their corresponding radical anions.^[^ ^180^ ^]^

The collapse of radicals **344a–e** leads to the reduction of the nitro group, forming nitroso intermediates **345a–e**. These intermediates then undergo intramolecular electrophilic aromatic substitution, resulting in the formation of hydroxyindoles **346a–e**. The authors remain uncertain whether this step occurs outside the metal's coordination sphere or if palladium actively facilitates the process. Reduction of **346a–e** by the palladium‐carbonyl complex ultimately yields the final indoles **158a** and **341a–d**, while simultaneously regenerating the active catalyst.^[^ ^179^ ^]^

### 9.9 Catalytic Synthesis of 2,3‐Substituted Indoles via α‐(o‐Chloro)aryl Ketones and (Hetero)aryl Nitriles

**Scheme 44:**
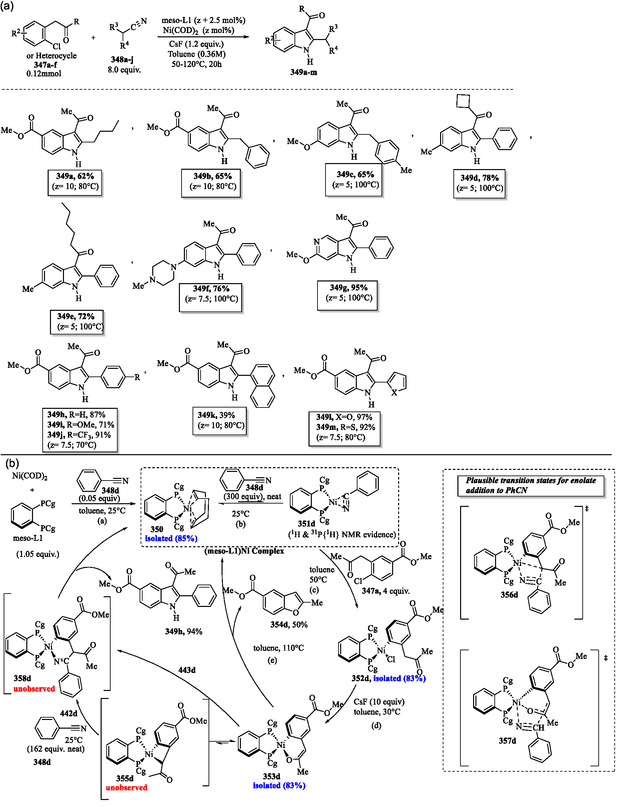
a) Catalytic synthesis of 3,2‐substituted N*H*‐indoles **349a–m** from α‐(*o*‐chloro)aryl ketones **347a–f** and (hetero)aryl nitriles **348a–j** and b) proposed mechanism.

MacMillan et al. reported the synthesis of 2,3‐substituted indoles **349a–m** via the reaction of α‐(*o*‐chloro)aryl ketones **347a–f** with (hetero)aryl nitriles **348a–j**, catalyzed by meso‐L1, a nickel complex bearing the meso‐PAd2‐DalPhos ligand (**Scheme** **44**a). Reaction control is crucial for directing the process toward indole formation, as it competes with the formation of benzofuran. The authors reported that the optimal conditions to suppress side‐product formation involve using CsF as the base associated with low temperatures. The reaction proceeded with good to excellent yields, with the highest yields observed for nitriles bearing electron‐withdrawing groups. These groups enhance the electrophilic character of the nitrile, facilitating the key step of nickel enolate addition.^[^ ^181^ ^]^

The authors proposed a mechanism that begins with the formation of (meso‐L1)Ni(COD) complex **350** by treating meso‐L1 with Ni(COD)_2_ in toluene. The process is facilitated by the addition of PhCN **348d** (5 mol% relative to Ni) as a traceless additive to promote ligand exchange, resulting in complex **350** with an isolated yield of 85%. However, when the reaction is conducted in a neat medium with an excess of PhCN (300 equiv.), complex **350** exists in equilibrium with phosphorus‐containing species **351d**, as confirmed by NMR spectroscopy data (^1^H and ^3^ ^1^P).

In the presence of aryl chloride electrophile **347a** in toluene, the Ni(0) complex **351d** undergoes oxidative addition to form the diamagnetic Ni(II) C—Cl complex 438 d, isolated in 83% yield. Treatment of **352d** with CsF at 30 °C induces HCl elimination, yielding the Ni(II) enolate species **353d** in 83% yield. The capability of **353d** to undergo C—O bond reductive elimination, thereby completing the benzofuran synthetic catalytic cycle, is confirmed by heating **353d** to a catalytically relevant temperature (110 °C). Analysis of the reaction mixture by gas chromatography‐flame ionization detection revealed the presence of compound **354g**, obtained in 50% yield. The authors reported that **353d** exists in equilibrium between its κ^2^‐C,O species and the κ^2^‐C,C α‐arylation intermediate **355d**. However, no ^1^H or ^3^ ^1^P{^1^H} NMR spectroscopic evidence was found to support the existence of this equilibrium leading to **355d** (Scheme 44b).^[^ ^181^ ^]^

Nonetheless, in neat PhCN at 25 °C, **355d** converts to *NH*‐indole **349h** with 94% isolated yield, accompanied by the formation of **358d**. The authors also suggest that the net 1,2‐migratory insertion of PhCN into **355d** generates nickel(II) iminyl species **358d** via **356d**, followed by rapid C(sp2)—N(sp2) bond reductive elimination and tautomerization, ultimately yielding **349h** and completing the *NH*‐indole synthetic catalytic cycle. However, due to the absence of spectroscopic evidence for **355d**, the authors also consider **357d** as a potential pathway for nitrile addition directly from **353d**. The authors proposed this mechanism based on reaction monitoring and kinetic studies, successfully isolating intermediates **352d** and **353d** (Scheme 44b).^[^ ^181^ ^]^

### 9.10 Ruthenium‐Catalyzed Synthesis of Substituted Indoles from Hydrazine‐Derived Enamines and Propargyl Alcohols

**Scheme 45:**
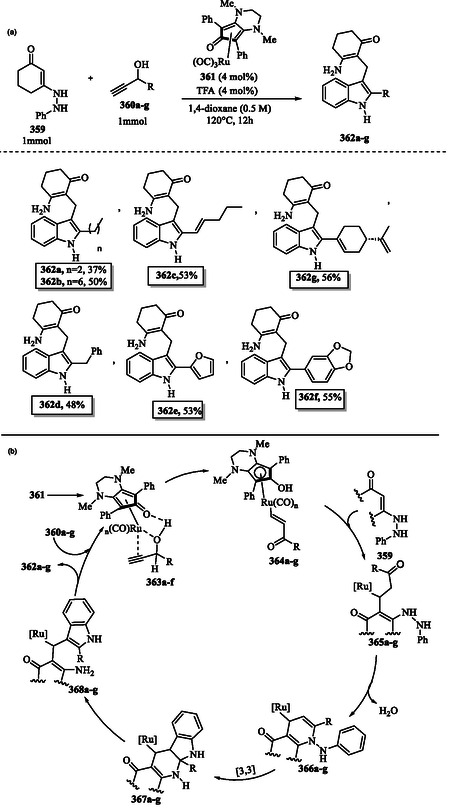
a) Ruthenium‐catalyzed synthesis of substituted indoles **362a–g** from hydrazinylenone **359** and secondary propargylic alcohols **360a–g** and b) proposed mechanism.

Skowaisa and Haak reported the ruthenium‐catalyzed synthesis of substituted indoles **362a–g** from hydrazinylenones **359** and secondary propargylic alcohols **360a–g** (**Scheme** **45**a). The use of diaminocyclopentadienone–ruthenium complex **361** in 1,4‐dioxane, together with trifluoroacetic acid (TFA) as an acidic additive, enabled the efficient synthesis of substituted indoles **362a–g** in good yields.^[^ ^182^ ^]^ The cascade reaction (Scheme 45b) begins with the activation of propargyl alcohols **360a–g** by the catalyst **361**, resulting in the formation of the π‐complexes **363a–f**. This step is followed by a 1,2‐hydrogen shift, generating the ruthenium alkenyl intermediates **364a–g** via a redox isomerization process. The Michael addition of hydrazinylenone **359**, followed by cyclocondensation, leads to the formation of dihydropyridine species **366a–g** via intermediate complexes **365a–g**. Next, a Fischer indole synthesis takes place, forming complexes **368a–g** via intermediates **367a–g**. Finally, reductive elimination releases the indole products **362a–g** and regenerates the catalytically active species.^[^ ^182^ ^]^

### 9.11 Synthesis of Cyclohepta[b]indoles and Cyclohepta[b]Indole‐Indoline Derivatives via Ring‐Closing Metathesis (RCM), Hydrogenation, and Acid‐Catalyzed Ring Expansion

**Scheme 46:**
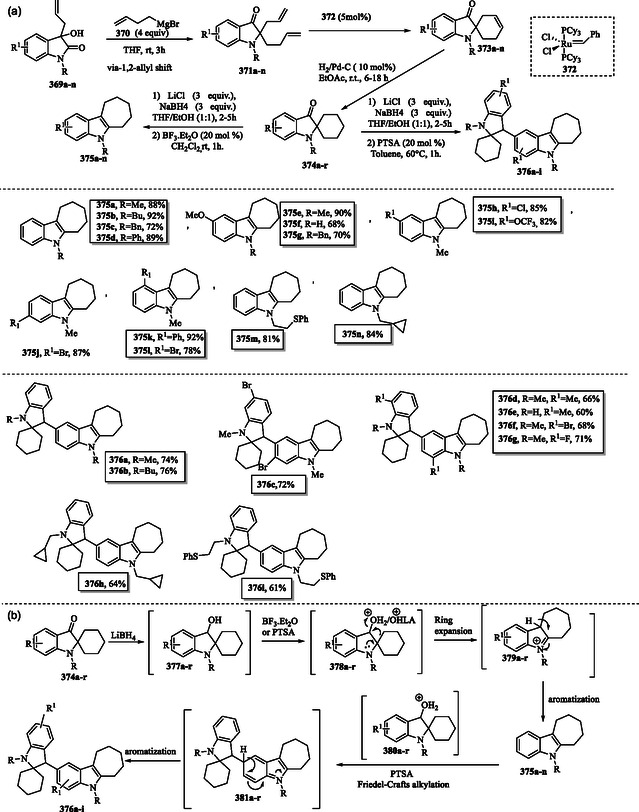
a) Synthesis of cycloheptaindoles **375a–n** and cycloheptaindole‐indoline conjugates **376a–I** and b) proposed mechanism.

Parui et al. developed an approach utilizing isatin derivatives **369a–n** as starting materials for the synthesis of cyclohepta[*b*]indoles **375a–n** and cyclohepta[*b*]indole‐ indoline derivatives **376a–i** (**Scheme** **46**a). 2‐Allyl‐2‐(but‐3‐enyl)‐3‐oxindole derivatives **371a–n** undergo ring‐closing metathesis (RCM) catalyzed by 5 mol% of the first‐generation Grubbs catalyst **372** in anhydrous CH_2_Cl_2_ at 45 °C, yielding spirocyclohexene‐3‐oxindole derivatives **373a–n**. Subsequently, catalytic hydrogenation of **373a–n** affords spirocyclohexane‐3‐oxindole compounds **374a–r**. In the next step, spirocyclohexane‐3‐oxindoles **374a–r** are reduced with NaBH_4_ in the presence of LiCl, followed by treatment with the Lewis acid BF_3_·Et_2_O in CH_2_Cl_2_, resulting in the formation of indole derivatives **375a–n**. Alternatively, when the reduced oxindoles **374a–n** are treated with the Brønsted acid *p*‐toluenesulfonic acid (PTSA, 20 mol%) in toluene at 60 °C, cyclohepta[*b*]indole‐indoline conjugates **376a–i** are obtained as the major products (Scheme 46a).^[^ ^183^ ^]^

The authors proposed a mechanism for the formation of two series of target compounds, **375a–n** and **376a–i**, from spirocyclohexane‐3‐oxindoles **374a–r** (Scheme 46b). The oxindoles **374a–n** are initially reduced using LiBH_4_, generated in situ from NaBH_4_ and LiCl, to afford intermediates **377a–r**. Subsequent protonation or coordination of **377a–r** with a Lewis acid, in the presence of an acid catalyst, facilitates the formation of **378a–r**, which then undergoes spontaneous 1,2‐bond migration to yield the ring‐expanded indolinium‐ions **379a–r**. Aromatization of **379a–r** leads to the formation of cyclohepta[*b*]indole derivatives **375a–n**. In the presence of PTSA, compounds **379a–r** undergo Friedel–Crafts alkylation with **380a–r** to form indolinium ions **381a–i**. Subsequent aromatization of **381a–i** yields the cyclohepta[*b*]indole‐indoline conjugate derivatives **376a–i**.^[^ ^183^ ^]^

### 9.12 Ruthenium‐Catalyzed Indole Formation from N‐Aryl‐2‐Aminopyridines and A‐Carbonyl Sulfoxonium Ylides

**Scheme 47:**
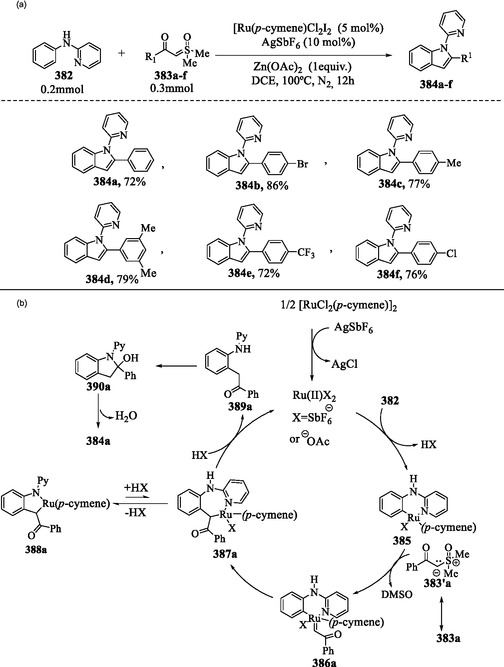
a) Ruthenium‐catalyzed synthesis of indoles **384a–f** from *N*‐aryl‐2‐aminopyridines **382** and α‐carbonyl sulfoxonium ylides **383a–f** and b) proposed mechanism.

Cui et al. proposed an efficient Ru(II)‐catalyzed synthesis of indoles **384a–f** via the intermolecular annulation of *N*‐aryl‐2‐aminopyridines **382** with sulfoxonium ylides **383a–f** (**Scheme** **47**a). This protocol afforded good yields under optimized conditions, utilizing silver hexafluoroantimonate (AgSbF_6_) as a cationic initiator, zinc acetate (Zn(OAc)_2_) as an additive, and 1,2‐dichloroethane (DCE) as the solvent, with the reaction carried out at 100 °C under a nitrogen atmosphere. This methodology was successfully applied to a diverse range of *N*‐aryl‐2‐aminopyridines **382** and sulfoxonium ylides **383a–f**, featuring both electron‐donating and electron‐withdrawing substituents.^[^ ^184^ ^]^

The proposed mechanism for the annulation of compound **382** with sulfoxonium ylides **383a–f** (Scheme 47b) initiates with the conversion of the dimeric precursor [Ru(*p*‐cymene)Cl_2_]_2_ into the active catalyst [Ru(II)X_2_] via anion exchange. Subsequently, the nitrogen atom of *N*‐aryl‐2‐aminopyridine **382** coordinates to the metal center, triggering cyclometalation and resulting in the formation of a six‐membered ruthenacycle intermediate **385**. Coordination of sulfoxonium ylide **383a** to the metal center, followed by DMSO elimination, generates the Ru(II) carbene intermediate **386a**. This intermediate then undergoes migratory insertion of the Ru–aryl bond into the activated carbene, resulting in the formation of the seven‐membered ruthenacycle intermediate **387a**. The authors suggest that a five‐membered cyclic complex **388a** may also form via deprotonation of **387a**. Protonolysis of the Ru—C bond by HX generates intermediate **389a** and regenerates the Ru(II)(*p*‐cymene) catalyst. Subsequently, an intramolecular nucleophilic attack of the imine on the carbonyl group forms intermediate **390a**, which then undergoes dehydration to yield the final product **384a**.^[^ ^184^ ^]^

### 9.13 Catalyzed Synthesis of Indole‐2‐Carboxylic Esters via Intramolecular Amidation of α‐Amidoacrylates

**Scheme 48:**
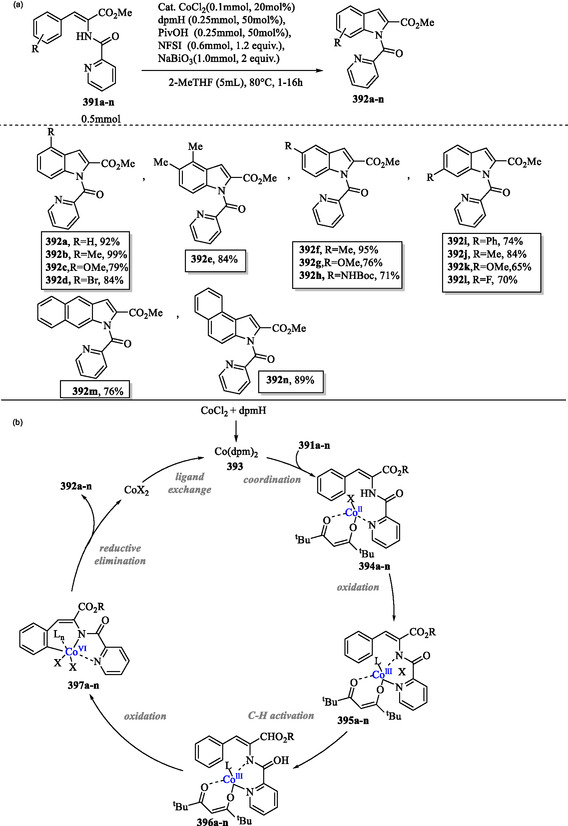
a) Catalyzed indole formation from intramolecular amidation of amidoacrylates **391a–n** and b) proposed mechanism.

Cizikov et al. developed an efficient catalytic protocol for synthesizing indole‐2‐carboxylic esters **392a–n**, achieving moderate to excellent yields via intramolecular amidation of amidoacrylates **391a–n** (**Scheme** **48**a).^[^ ^185^ ^]^ The authors performed mechanistic investigations through a series of control experiments, cyclic voltammetry analyses, and computational studies (Scheme 48b).

The process involves the coordination of the in situ generated Co(dpm)_2_ catalyst **393**, formed from CoCl_2__2_ and the dipivaloylmethane ligand, to the α‐amidoacrylate substrates **391a–n**. Oxidation of the Co(II) complexes **394a–n** produces Co(III) species **395a–n**, which then undergo C(sp^2^)—H bond cobaltation to form Co(III) complexes **396a–n**. Oxidation of the Co(III) intermediates **396a–n** to high‐valent Co(IV) species produces complexes **397a–n**, which rapidly undergo reductive elimination to release products **392a–n** and regenerate the Co(II) species. Following ligand exchange, the Co(II) species re‐enters the catalytic cycle, thus ensuring continuous turnover.^[^ ^185^ ^]^

## 10 Conclusion

Indole stands out as one of the most versatile and important heterocyclic systems in modern chemistry. As detailed in this review, this privileged scaffold is present not only in the structural frameworks of natural products but also in numerous synthetic drugs with proven antitumor, antimicrobial, and anti‐inflammatory activities. Beyond medicinal applications, indole derivatives exhibit remarkable potential in materials science, serving as corrosion inhibitors, antifouling agents in marine coatings, and fluorescent probes for the selective detection of metal ions.

Advances in synthetic methodologies have been transformative. From classical approaches like the Fischer and Bischler syntheses to modern strategies involving metal‐catalyzed (Pd and Ru) C—H activation and metal‐free photoredox catalysis, the synthesis of indole derivatives has become significantly more efficient and sustainable. These developments have expanded access to more complex structural analogs in the pharmaceutical and agrochemical fields.

Looking ahead, several key challenges persist. The development of greener, more selective, and cost‐effective synthetic strategies remains a top priority, especially for large‐scale production. Furthermore, gaining a deeper understanding of the biological activities and mechanisms of action of indole‐based compounds is crucial to fully harness their therapeutic potential. Despite these challenges, the progress achieved so far in both synthetic innovation and the broad range of applications highlights the fundamental role of indole chemistry in drug discovery and materials science.

This review serves as a guide for researchers, addressing the biosynthesis of key indoles, the relevance of the indole nucleus in medicinal chemistry, the optimization of traditional synthetic routes, and the exploration of new applications in materials science, further reinforcing the essential role of indoles in scientific research and industrial development.

## Conflict of Interest

The authors declare no conflict of interest.

## Author Contributions

**Raphael Silva Moratório de Moraes** conducted the literature search, formatted the text and wrote the methodology section on indole‐based reactions involving catalysts. **Ana Beatriz Mestre Botelho**, and **Gabriel Tavares de Almeida Pinto** contributed to the literature search and writing in the field of materials chemistry. **Searitha Couto Rodrigues** performed the literature search, selected appropriate synthetic methodologies, and organized the bibliography. **Maria Tereza Miranda Martins** was responsible for the literature search, selection of synthetic methodologies, formatting of the text and scheme structures, and organization of the bibliography. **Deivid Lucas Alves Soares**, **Camille Cardoso Cruz**, and **Aline de Almeida Pinto** contributed to the review of the text, preparation of the scheme structures, and literature research. **Flaviana Rodrigues Fintelman Dias** wrote the methodology section involving green chemistry. **Patricia Dias Fernandes** wrote the section on applications of indoles. **Anna Claudia Cunha** wrote the review, conducted literature search and selection, supervised the work, and contributed to the review and conceptualization of the study.
